# Glyoxal is a superior fixative to formaldehyde in promoting antigenicity and structural integrity in murine cardiac tissues

**DOI:** 10.1016/j.jmccpl.2025.100454

**Published:** 2025-05-11

**Authors:** Allen C.T. Teng, Dev Mehangrey, Ava Vandenbelt, Karl Vearncombe, Justin D. Callahan, Priya Mistry, Wenping Li, Cristine J. Reitz, Omar Hamed, Madison Roche, Uros Kuzmanov, Jason E. Fish, Slava Epelman, Anthony O. Gramolini

**Affiliations:** a Translational Biology and Engineering Program, Ted Rogers Centre for Heart Research, Canada; b Department of Physiology, University of Toronto, Canada; c Department of Laboratory Medicine and Pathobiology, University of Toronto, Canada; d Toronto General Hospital Research Institute, University Health Network, Canada; e Department of Immunology, University of Toronto, Canada; f Peter Munk Cardiac Centre, University Health Network, Toronto, ON, Canada

**Keywords:** Fixatives, Immunofluorescence, Crosslinked immunoprecipitation-mass spectrometry, Cardiac biochemistry

## Abstract

**Background:**

Immunofluorescence (IF) is an essential technique for evaluating histological and biochemical changes in tissue specimens. A critical step in IF is sample fixation, typically achieved using formaldehyde-based fixatives, such as 4 % paraformaldehyde (PFA) or 10 % formalin. However, these fixatives are prone to over-fixation, which can alter antigenicity and promote artifacts. This study investigated glyoxal, a two‑carbon dialdehyde, as a potential alternative fixative for murine cardiac tissues for IF and crosslinking immunoprecipitation-mass spectrometry (xIP-MS) applications.

**Methods:**

Various concentrations and fixation durations of glyoxal were compared with 4 % PFA. Tissue structural integrity was assessed using Hematoxylin and Eosin (H&E) staining, while antigen preservation in cardiomyocytes was evaluated through fluorescent microscopy. Immunofluorescence of cardiac resident cells, including cardiac fibroblasts, smooth muscle cells, and endothelial cells were also investigated. xIP-MS assays were carried by phospholamban (PLN) immunoprecipitation in glyoxal-fixed mouse hearts, followed by mass spectrometry analysis.

**Results:**

Glyoxal showed comparable preservation of cardiac tissue architecture and myofiber integrity to PFA, but with superior antigen retention and protein detection. Fluorescent imaging was performed for sarcoplasmic reticulum markers (SERCA2 and PLN), intercalated disc proteins (N-Cadherin and Cx43), and contractile proteins (F-Actin and MyHC). Quantitative image analysis confirmed that glyoxal enhanced antibody penetration in thicker tissues (30 μm) and maintained the antigenicity of various cardiac resident cell markers. Glyoxal fixation allowed for xIP-MS by lightly crosslinking PLN with its associated protein complexes, enabling the identification of novel PLN-interacting proteins in mouse hearts.

**Conclusion:**

Our findings underscore the utility of glyoxal as a superior alternative to PFA in cardiac biochemistry research, offering improvements in the preservation of tissue morphology, antigen detection, and protein complex conservation in murine cardiac tissues.

## Highlights

• Glyoxal enhances IF signal, co-localization, and reduces staining artifacts.
• 3 % glyoxal preserves heart structure; 9 % disrupts histology and architecture.
• Glyoxal improves antibody access to heart-resident cells via light crosslinking.
• Light glyoxal crosslinking enables better protein extraction for cardiac xIP-MS.

## Graphical abstract

**Figure:**
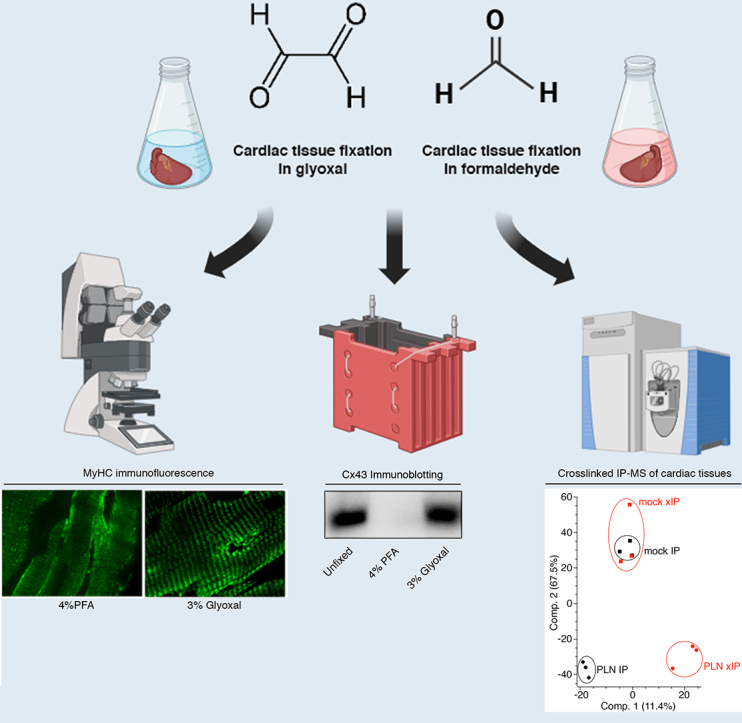

## 1 Introduction

Immunofluorescence (IF) and immunoprecipitation (IP) are essential techniques for studying histological and biochemical changes in tissues and cells. A critical step in both methods involves the use of fixatives, which preserve tissue structure and stabilize macromolecules in their native states [1]. Formaldehyde (HCOH), a one‑carbon aldehyde, has traditionally been the standard fixative, typically applied as 4 % paraformaldehyde (PFA) or 10 % formalin [1]. These fixatives work by forming covalent methylene (-CH2-) crosslinks between amino groups on adjacent proteins and nucleic acids [2], effectively locking proteins in place. However, this crosslinking process can sometimes lead to over-fixation and conformational changes, which may interfere with antibody-antigen binding, thereby reducing both signal intensity and specificity in IF [1]. Although antigen retrieval and enzymatic digestion techniques are commonly used to reverse crosslinking, they often yield inconsistent results due to harsh conditions (e.g., elevated temperatures and pressure) and the risk of unintended antigen degradation [1].

Glyoxal (OCHOCH), a two‑carbon dialdehyde, may be a promising alternative to conventional fixation methods. Konno et al. investigated glyoxal fixation in murine brain tissues and found that it improved antibody penetration and immunoreactivity, enabling accurate detection of neuronal proteins that were otherwise masked by formaldehyde fixation [3]. Similarly, Richter et al. demonstrated that glyoxal outperformed traditional fixatives in immunostaining and super-resolution microscopy across several cell lines [4]. Glyoxal has proven effective across a wide range of cell types, including zebrafish and fruit fly embryos [5,6]. These findings suggest that glyoxal may be a superior fixative to formaldehyde for tissues and cells, though its efficacy in cardiac tissues and cardiomyocytes remains unexplored. In this study, we evaluated the impact of glyoxal fixation on cardiac tissue integrity and antigen immunogenicity in comparison to 4 % PFA. Our analysis focused on the fluorescence imaging of both cardiomyocyte organelles and cardiac resident cell types in mouse hearts. We also examined the applicability of glyoxal in crosslinking immunoprecipitation-mass spectrometry (xIP-MS) from mouse hearts. Our results indicate that glyoxal is a more effective fixative than PFA in mouse hearts, reducing background noise, enhancing antigenicity, and improving antigen accessibility in immunohistology. Glyoxal fixation also enables whole heart protein-protein interactome investigations through xIP-MS.

## 2 Materials and methods

### 2.1 Chemicals

Paraformaldehyde (Sigma-Aldrich, 158,127), acetic acid (Sigma-Aldrich, 695,092), 40 % glyoxal (Sigma-Aldrich, 128,465), Mayer's Hemotoxylin (Sigma-Aldrich, MHS32-1 L), Eosin Y (Sigma-Aldrich, 119,830-25G), 2-methylbutane (Sigma-Aldrich, 270,342), Hoescht 33,342 nucleic acid stain (Invitrogen, H3570), rectangular No.1 cover slips (VWR, 48393–081), ethanol (Fisher, A405P4), Permount Mounting Media (Fisher, SP15–100), Superfrost Plus Microscope Slides (Fisher, 1,255,015) and Optimal Cutting Temperature Compound (OCT, Fisher, 23–730-571).

### 2.2 Antibodies

Antibodies, commercial suppliers, and dilution ratios for immunofluorescence are listed below. PLN (1:250 Invitrogen, MA3–922), SERCA2 (1:500, ab3625, Abcam), Phalloidin (1:400, Fisher, A12380), Myosin Heavy Chain-1 (1:5, DSHB, MF-20), Periostin (1:400, Abcam, ab14041), Smooth muscle actin (1:200, Cell Signaling Technology, cs48938), Tubulin III (1:1000, Abcam, ab18207), CD31 (1:100, BD Pharmingen 553,370), VE-Cadherin (1:100, Santa Cruz, sc-9989), N-Cadherin (1:400, Cell Signaling, 81,673), Connexin-43 (1:100, Sigma-Aldrich, ZRB1179), anti-Mouse Alexa 488 (1:1000, Invitrogen, A28175), anti-Rabbit Alexa 568 (1:500, Invitrogen, A-11011).

### 2.3 Fixative solution preparation

To prepare 4 % PFA, 8 g of paraformaldehyde was added to 100 mL of ddH_2_O. 1 mL of NaOH was added dropwise and stirred gently on a heating block at 60 °C until homogenous. To prepare glyoxal fixative solutions, each component was thoroughly mixed at room temperature according to Table 1. Glyoxal solutions were light-protected and stored at 4 °C for a maximum of one month.

**Table 1:** Composition of buffers used in this study.

To form a 100 mL buffer solution	40 % solution of glyoxal	100 % ethanol	100 % acetic acid	Phosphate buffered saline
3 % glyoxal	7.5 mL			92.5 mL
3 % glyoxal/ 0.8 % acetic acid	7.5 mL		0.8 mL	91.7 mL
3 % glyoxal/ 20 % ethanol	7.5 mL	20 mL		72.5 mL
3 % glyoxal/ 20 % ethanol/ 0.8 % acetic acid	7.5 mL	20 mL	0.8 mL	71.7 mL
9 % glyoxal / 8 % acetic acid	22.5 mL		8 mL	69.5 mL

### 2.4 Cardiac tissue harvest, cryopreservation, cryo-sectioning

C57BL/6 J mice were used for this study. Mice were CO_2_ euthanized and subjected to cervical dislocation at 6–8 weeks of age. Mice were laid in a supine position, and their chest cavity was opened to locate the heart. A small incision was made between the right atrium and ventricle. 5 mL of 1×PBS and fixative solutions were injected through the left ventricle for perfusion. Hearts were surgically removed and placed in the corresponding fixative solution overnight at 4 °C. Next day, hearts were washed once with 1× PBS and then submerged in 15 % (*w*/*v*%) sucrose solution overnight at 4 °C. The same step was repeated with 30 % (w/v%) sucrose solution. To cryopreserve mouse hearts, samples were placed in cryomolds, embedded in OCT, then submerged in dry ice-chilled 2-methylbutane to allow for quick and even OCT freezing. Cryosections at 5 μm and 30 μm were prepared on a Microm HM-500 OM cryostat, and sections were placed on Superfrost Plus Microscope Slides. Tissue sections were stored at -80 °C until use.

### 2.5 H&E staining and quantification

Eosin working solution was made in house. 1 % eosin stock solution was prepared by combining 10 g of stock eosin Y, 200 mL of ddH_2_O, and 800 mL of 95 % ethanol. The 0.25 % working solution was prepared by combining 250 mL of 1 % eosin stock solution, 750 mL of 80 % ethanol, and 5 mL of glacial acetic acid. Tissues were first stained with hemotoxylin solution for 16 min, then washed in warm, running water for 3.5 min. Slides were dipped 10 times into 95 % ethanol, counterstained in the 0.25 % eosin solution for 1 min, washed twice for 5 min with 95 % ethanol, and left to air dry. No. 1 rectangular cover slips were applied using a toluene-based mounting media, and images were captured on a Zeiss spinning disc confocal microscope (Zeiss Observer Z1). Image analysis was completed on ImageJ (NIH) using the following workflow: 1) convert to grey scale (*Image ➔ Type ➔ RGB Stack ➔ Green Channel)*, 2) define periphery of myofibers using the green channel, 3) determine areas of interest with thresholding (*Image ➔ Adjust ➔ Threshold ➔ Set).*

### 2.6 Immunofluorescence, calculations for signal-to-noise ratios and mean fluorescent intensity (MFI) in z-stacked images

Frozen cardiac tissues were washed twice with ice-cold 1× PBS for 3 min to remove residual OCT. Tissue sections were permeabilized (PBS; pH 7.4, 0.5 % (*v*/v) Triton X-100, 0.2 % (v/v) Tween 20) then blocked (Permeabilization solution +5 % FBS), each for 1 h at room temperature in a humidified chamber. Tissues were then incubated with primary antibodies overnight at 4 °C in a humidified chamber. The next day, slides were washed 3 times (3 min per wash) with ice-cold 1× PBS, then incubated with secondary antibodies for 1 h at room temperature, in the dark. All antibodies were diluted in blocking buffer (1× PBS, 0.1 % Tween-20, 5 % (*w*/*v*%) BSA) according to *Antibodies* subsection. Tissues were washed 3 times with 1× PBS (3 min per wash) and counterstained with 1 μg/mL Hoechst 33342 (#4082, Cell Signaling Technology) for 5 min at room temperature, in the dark. Tissues were washed 3 more times with 1× PBS (3 min per wash), No. 1 rectangular cover slips were applied using a toluene-based mounting media, and images were captured on a confocal microscope (Zeiss Observer Z1, Zeiss Axio Imager M2, or a Nikon A1R Confocal Microscope). ImageJ (NIH) was used to evaluate signal-to-noise ratios. Individual channels were split into Greyscale (Image ➔ Colour ➔ Split Channels), colours reassigned (Image ➔ Lookup Tables) and merged together (Image ➔ Colour ➔ Merge Channels). An orthogonal line was drawn across sarcomere or sarcoplasmic reticulum staining then registered in ROI manager (Analyze ➔ Tools ➔ ROI Manager). Grayscale analysis was then performed (Analyze ➔ Plot ➔ Profile) on oscillation patterns of sarcomeres. The peak and valley values of each oscillate were registered as signal and noise, respectively, while their division determined the signal-to-noise ratio. MFI calculation is described below. A *Z*-stack is a series of images taken at different depths along the z-plane, while the x and y coordinates remain constant. Each component image of a Z-stack (Fig. 5) was loaded into ImageJ (File ➔ Import ➔Image Sequence) and converted to grey scale (Image ➔ Type ➔ 8-bit). Signal intensity was then readjusted (Image ➔ Adjust ➔ Threshold), so that only sarcomere signals turned red in the software. Signal intensity and area were then measured (Analyze ➔ Measure) and MFI was calculated using $\frac{\mathrm{Signal Intensity}}{\mathrm{Signal Area}}$.

### 2.7 SDS-PAGE electrophoresis, in-gel Coomassie blue staining, and immunoblotting

Following an overnight fixation, murine hearts were washed twice with 1× PBS, homogenized in liquid nitrogen with mortar and pestle. Proteins were subsequently extracted in freshly prepared radioimmunoprecipitation assay (RIPA) buffer (10 mM Tris-HCl; pH 8.0, 1 mM EDTA, 0.5 mM EGTA, 1 % Triton X-100, 0.1 % sodium deoxycholate, and 0.1 % SDS, 150 mM NaCl), supplemented with 1× protease inhibitors (Roche). Protein concentration was determined by Bradford assays and 50 μg of total proteins were loaded for SDS-PAGE electrophoresis. Experimental procedures for SDS-PAGE electrophoresis, Coomassie blue staining [7], and immunoblotting [8] were previously reported. Antibodies include anti-phospholamban antibody (1:1000 dilution, MA3–922, Invitrogen), anti-SERCA2 antibody (1:1000, 2A7-A1, Invitrogen), anti-N-Cadherin (1:1000, D4R1H, Cell Signaling Technology), anti-Connexin43 antibody (1:500, ZRB1179, Sigma-Aldrich), anti-α-Actin (1:250, JLA20, Developmental Studies Hybridoma Bank).

### 2.8 Immunoprecipitation and mass spectrometry

Mouse hearts fixed overnight in 3 % glyoxal were washed with ice-cold 1× PBS 3 times. Unfixed, snap frozen hearts were included as controls. Protein lysates were extracted as previously described with modification [9]. Mouse hearts were pulverized in mortar and pestle in liquid nitrogen. Cardiac lysates were extracted with RIPA buffer, supplemented with protease inhibitors (Sigma), and were incubated on ice for 10 min. Insoluble fractions were spun down at 12,000 ×*g* for 10 min and soluble fractions were transferred to a new microcentrifuge tube. Protein concentration was measured by Bradford assays. 1 mg of soluble proteins in 1 mL final volume (topped up with IP buffer) were precleared with 50 μL of protein A/G beads (Invitrogen) for 30 min at 4 °C. Precleared lysates were incubated with 2 μg of PLN antibody (Invitrogen) overnight at 4 °C on an end-to-end rotator. Next day, antibody-lysate mix was incubated with protein A/G beads for 1 h a 4 °C and immobilized proteins were washed with 50 mM ammonium bicarbonate 5 times. On-bead digestion was performed with trypsin (Promega, V5280) overnight at 37 °C. Tryptic peptides were loaded on C18 tips (Evosep, EV-2001) using manufacturer instructions and subjected to in-line chromatography using the Evosep One (EV-1000) instrument 15SPD LC protocol. Here, an 88-min gradient was used with the Endurance 15 cm analytical column (EV-1106) and a stainless-steel emitter (EV-1086), with a spray voltage of 1.9 kV. For DIA MS data acquisition, the Q exactive Plus instrument was used, with each cycle involving one full MS scan (at 70,000 resolution) followed by 30 MS/MS scan windows (at 17,500 resolution) covering a mass range from 400 to 951 *m*/*z*. The AGC target was set to 3e6 for full MS scans and 5e5 for MS/MS scans, with a maximum injection time of 200 ms for full MS scans and a normalized collision energy of 27 for MS/MS scans. The isolation windows were set to 20 m/z with 1 m/z overlap between scan windows. Generated RAW files were searched using the DIA-NN 1.81 search software. The data was searched against an DIA-NN generated in silico spectral library using the canonical and isoform protein sequence FASTA files from UNIPROT (UP000000589, February 2024 release). One missed cleavage and one variable modification (methionine oxidation) were allowed with carbamidomethylation of cysteines set as a fixed modification. Allowed charges were set 2 to 4 and precursor mass range was set to 350–950 *m*/*z*. The output was filtered based on a 0.01 precursor FDR, with the ‘match between runs (MBR)’, ‘unrelated runs’ and ‘use isotopologues’ features enabled. Quantification and cross-run normalisation parameters were set to ‘Robust LC (high precision)’ and ‘RT & signal-dep’, respectively. Mass accuracy and scan window were determined by DIA-NN built in features. Search data were imported into Perseus (v.1.6.2.1) for analysis. First, filter rows based on categorical column to exclude proteins identified by site, matching to the reverse database or contamination. Minimal valid value was at 3. Next, data were transformed to logarithmic scale (e.g., Log2(x)) and data distribution was confirmed by histograms and missing values were imputed. PCA plot was generated. Missing values were replaced from normal distribution as per Perseus default setup. For unsupervised hierarchical heatmap analysis, student's *t*-test was performed with 0.05 FDR cutoff and 0.1 standard variant. Functional analyses (Gene Ontology and Kyoto Encyclopedia Genes and Genomes) of significantly enriched proteins in IP and xIP groups were performed with ShinyGO (version 0.81, South Dakota State University) *Mus musculus* database at 0.05 FDR cut-off as previously reported [10].

## 3 Results

### 3.1 Evaluating glyoxal's impact on cardiac architecture

**Fig. 1:**
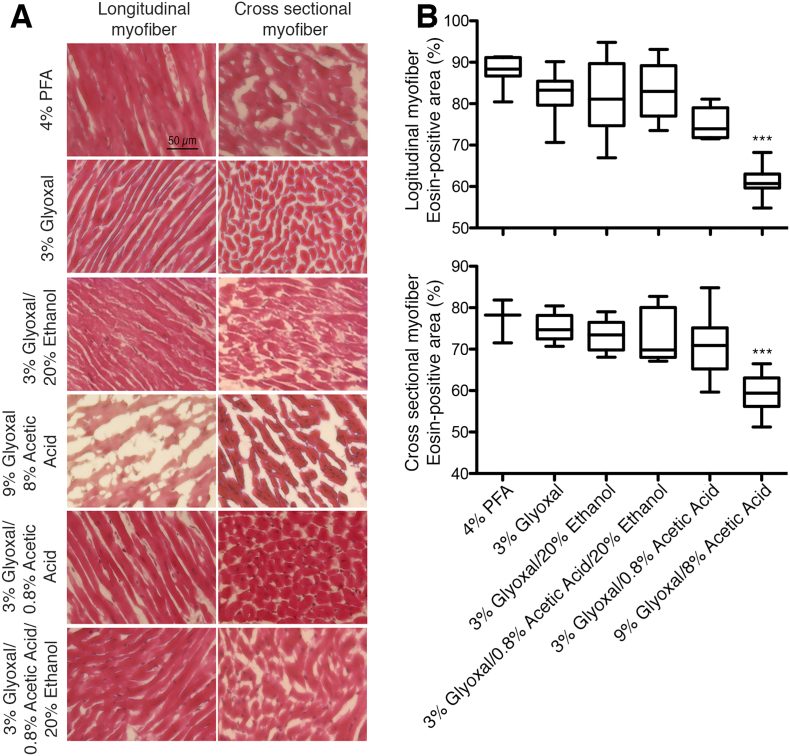
Hematoxylin and eosin staining of murine cardiac tissues in varying fixative conditions (A) Representative longitudinal and cross-sectional murine tissues shows no significant differences in myofiber structure among all fixatives, except 9 % glyoxal/ 8 % acetic acid. Scale bar = 50 μm. (B) Box plots visualizing the quantification of eosin-positive myofibers in fixative solutions. A significant reduced myofibers were found in 9 % glyoxal/8 % acetic acid. *n* > 15 images per fixative from 3 independent cardiac tissues. All box plots in the figure represent mean ± SEM. One-way ANOVA with Dunnett's post-hoc analysis was performed to assess the statistical significance. ***, *p* < 0.001.

This study is the first to assess the crosslinking properties of glyoxal as a fixative in cardiac tissues. Hematoxylin and eosin (H&E) staining was performed to confirm the preservation of cardiac structure and myofiber integrity following glyoxal fixation. Five different glyoxal-based fixatives were included in the investigation: 3 % glyoxal, 3 % glyoxal/20 % ethanol, 3 % glyoxal/0.8 % acetic acid/20 % ethanol, 3 % glyoxal/0.8 % acetic acid, and 9 % glyoxal/8 % acetic acid [3]. As shown in Fig. 1A, most glyoxal fixation conditions preserved cardiac tissue architecture similarly to 4 % PFA, as indicated by comparable eosin-positive areas across fixation conditions in both cross-sectional and longitudinal myofibers. However, one condition—9 % glyoxal +8 % acetic acid—resulted in myofibril shrinkage and increased inter-myofibrillar space (Fig. 1A). Quantitative H&E analysis confirmed this observation, revealing a significant reduction in eosin-positive myofibrils with the 9 % glyoxal +8 % acetic acid fixation compared to PFA-fixed tissues (Fig. 1B, *p* < 0.001; 87.97 ± 1.26 % in 4 % PFA vs. 61.15 ± 1.26 % in 9 % glyoxal/8 % acetic acid). These findings demonstrated that low concentrations (3 %) of glyoxal effectively preserved cardiac myofibril structure.

### 3.2 Evaluating glyoxal's impact on sarcoplasmic reticulum (SR) immunostaining

**Fig. 2:**
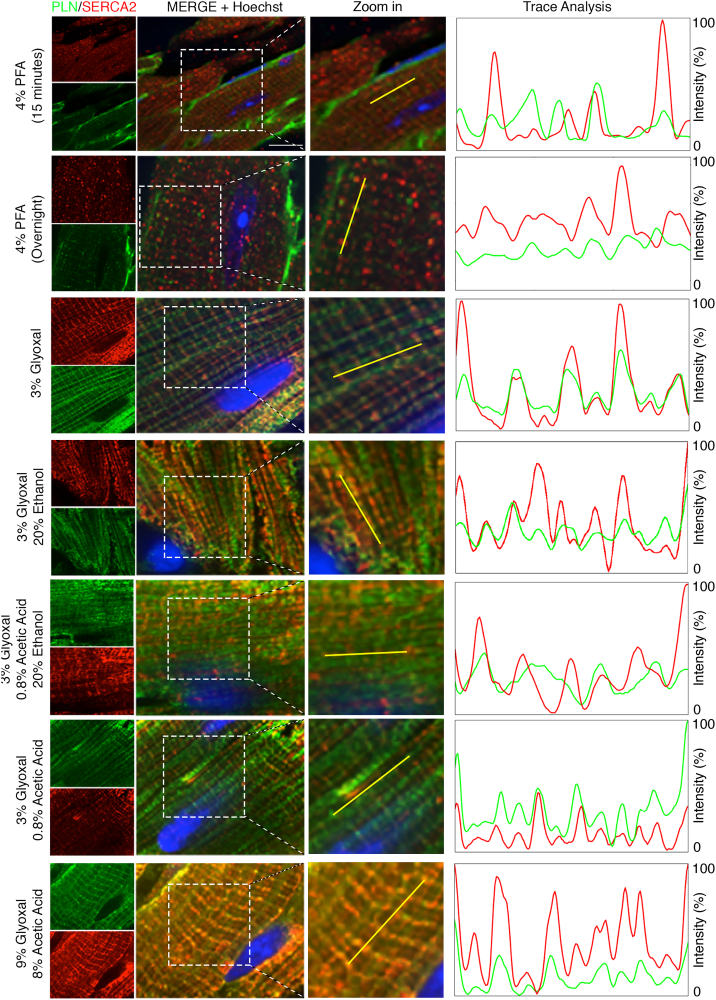
Immunofluorescence of SERCA2 and PLN in mouse hearts. Representative immunofluorescent images of sarcoplasmic reticulum membrane proteins PLN (green) and SERCA2 (red) in murine cardiac tissues. Scale bar = 20 μm. Right panels, trace analyses of PLN and SERCA2 signals in murine cardiac tissues with fixatives. PLN and SERCA2 signals had the best alignment in 3 % glyoxal-fixed cardiac tissues, followed by 3 % glyoxal/0.8 % acetic acid and 9 % glyoxal/8 % acetic acid. (Gly, glyoxal. EtOH, ethanol. AA, acetic acid. (For interpretation of the references to colour in this figure legend, the reader is referred to the web version of this article.)

**Supplementary Fig. 1:**
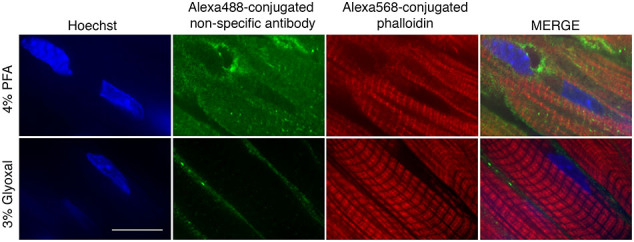
Quantification of signal-to-noise (S/N) in Fig. 2, Fig. 3, Fig. 4. (A) PLN fluorescent S/N ratio on the left and SERCA2 fluorescent S/N ratio on the right. *n* > 20 images per fixative from 3 independent cardiac tissues. All dot plots in the figure represent mean ± SEM. One-way ANOVA with Dunnett's post-hoc analysis was performed to assess the statistical significance. *, *p* < 0.05; ***, *p* < 0.001. (B) N-Cad fluorescent S/N ratio on the left and Cx43 fluorescent S/N ratio on the right. n > 20 images per fixative from 3 independent cardiac tissues. All box plots in the figure represent mean ± SEM. One-way ANOVA with Dunnett's post-hoc analysis was performed to assess the statistical significance. **, *p* < 0.01; ***, p < 0.001.Gly. (C) F-Actin fluorescent S/N ratio on the left and MyHC fluorescent S/N ratio on the right. n > 20 images per fixative from 3 independent cardiac tissues. All dot plots in the figure represent mean ± SEM. One-way ANOVA with Dunnett's post-hoc analysis was performed to assess the statistical significance. *, p < 0.05; ***, p < 0.001 determined by One-Way ANOVA with Dunnett's test.

We next sought to evaluate their impact on the antigenicity of proteins within dense organelles. To this end, we examined the immunofluorescent patterns of sarcoplasmic reticulum (SR) proteins phospholamban (PLN) and sarco(endo)plasmic reticulum Ca^2+^ ATPase (SERCA2) in murine cardiac tissues. In sections fixed with 4 % PFA, both 15-min and overnight fixation led to a stronger PLN signal at the plasma membrane (PM) compared to the SR membrane (Fig. 2A) which has been a known potential artifact of over-fixation. In contrast, no PM staining was observed in glyoxal-fixed cardiac tissues. Interestingly, while SERCA2 staining in 4 % PFA-fixed tissues appeared as puncta, glyoxal fixation revealed clearer longitudinal and transverse SR staining patterns for both PLN and SERCA2 (Fig. 2). To further assess the alignment of PLN and SERCA2, we analyzed RGB plots. As shown in the trace analysis panel of Fig. 2, minimal alignment was observed in tissues fixed overnight with 4 % PFA, while improved alignment was seen in sections fixed for 15 min with 4 % PFA. Among all the samples, those fixed with 3 % glyoxal exhibited the best alignment for both PLN and SERCA2, followed by tissues fixed with 3 % glyoxal/0.8 % acetic acid. Tissues treated with 3 % glyoxal/20 % ethanol, 3 % glyoxal/0.8 % acetic acid/20 % ethanol, or 9 % glyoxal/8 % acetic acid showed a lower signal-to-noise ratio (Supp. Fig. 1A, left panel, 1.16 ± 0.01 for SERCA2 and 1.15 ± 0.01 for PLN in overnight 4 % PFA, *p* < 0.001; 1.7 ± 0.02 for SERCA2 and 1.49 ± 0.03 for PLN in 3 % glyoxal/20 % ethanol, p < 0.001; 1.43 ± 0.02 for SERCA2 and 1.23 ± 0.01 for PLN in 3 % glyoxal/20 % ethanol/0.8 % acetic acid, *p* < 0.05; 1.68 ± 0.01 for SERCA2 and 1.44 ± 0.02 for PLN in 3 % glyoxal/0.8 % acetic acid, p < 0.001), suggesting that the presence of ethanol and/or acetic acid might have impaired antigen-antibody interactions. In contrast, tissues fixed with 3 % glyoxal displayed the most intact SERCA2 and PLN staining patterns, as well as the highest signal-to-noise ratios (Supp. Fig. 1A, right panel, 2.13 ± 0.01 for SERCA2, 1.68 ± 0.01 for PLN, p < 0.001) among all fixation conditions.

### 3.3 Evaluating glyoxal's impact on intercalated disc (ICD) immunostaining

**Fig. 3:**
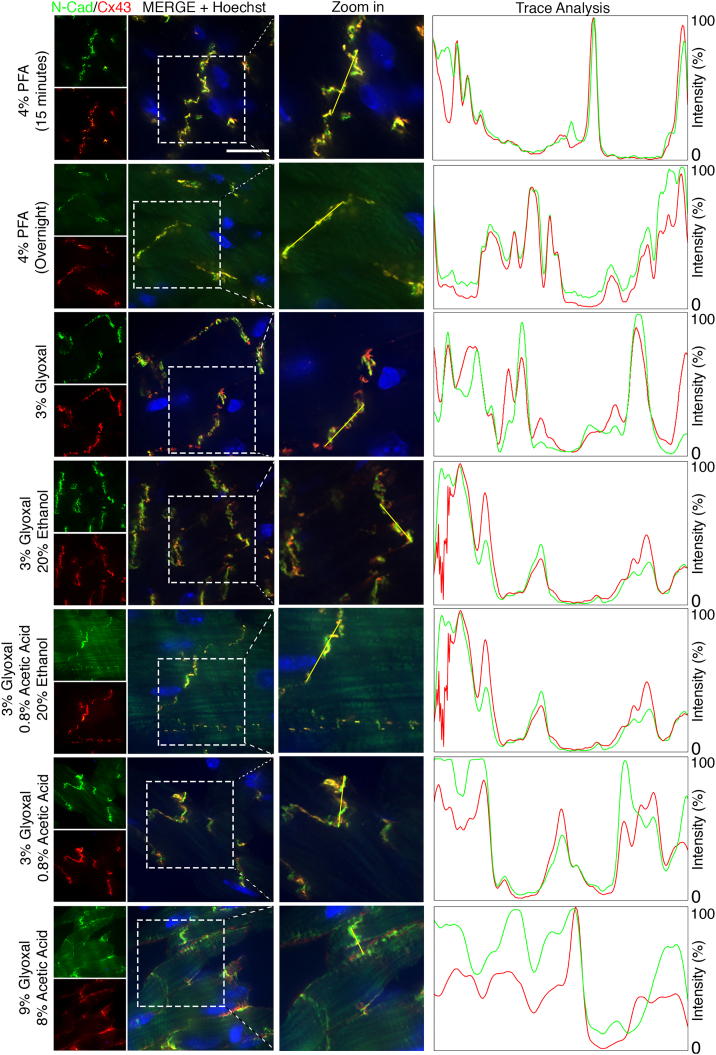
Immunofluorescence of Cx43 and N-Cadherin in mouse hearts. Representative immunofluorescent images of ICD proteins N-Cadherin (green) and Cx43 (red) in murine cardiac tissues with proposed fixatives. Scale bar = 20 μm. Zoom in views of the ICD images in Fig. 4A. N-Cadherin and Cx43 signals were juxtaposed in the hearts fixed with 3 % glyoxal or 3 % glyoxal/20 % ethanol, but were aligned in other fixatives. Right panels, trace analyses of signals in with fixatives. (Glyoxal. EtOH, ethanol. AA, acetic acid. (For interpretation of the references to colour in this figure legend, the reader is referred to the web version of this article.)

The ICD is essential for the structure and function of cardiomyocytes, and considerable efforts have been made to visualize ICD protein complexes, including adherens junctions, desmosomes, and gap junctions [8,11]. To investigate whether glyoxal enhances ICD imaging, we performed IF staining for adherens junctions (N-Cadherin) and gap junctions (Connexin43, Cx43). Cardiac tissues fixed with 4 % PFA for 15 min displayed a typical ICD staining pattern, with both N-Cadherin and Cx43 localized at the ICD between adjacent myocytes (Fig. 3A). Notably, Cx43 fully co-localized with N-Cadherin at the ICD, though some N-Cadherin signal was observed independently (Fig. 3A). In contrast, overnight fixation with 4 % PFA resulted in reduced ICD signal intensity and significantly increased background noise, suggesting over-fixation (Supp. Fig. 1B, left panel, N-Cad S/N ratio: 2.19 ± 0.19 for 15 min 4 % PFA, 0.70 ± 0.08 for overnight 4 % PFA, 3.41 ± 0.32 3 % for glyoxal, 2.70 ± 0.13 3 % for glyoxal/20 % ethanol, 0.57 ± 0.13 3 % for glyoxal/0.8 % acetic acid/20 % ethanol, 0.59 ± 0.06 for 3 % glyoxal/0.8 % acetic acid, 0.62 ± 0.07 for 9 % glyoxal/0.8 % acetic acid; Cx43 S/N ratio: 2.37 ± 0.19 for 15 min 4 % PFA, 0.90 ± 0.32 for overnight 4 % PFA, 3.15 ± 0.43 for 3 % glyoxal, 2.16 ± 0.51 for 3 % glyoxal/20 % ethanol, 1.80 ± 0.57 for 3 % glyoxal/0.8 % acetic acid/20 % ethanol, 1.19 ± 0.61 for 3 % glyoxal/0.8 % acetic acid, 1.21 ± 0.66 for 9 % glyoxal/8 % acetic acid). Similar issues were observed in tissues fixed with 3 % glyoxal/0.8 % acetic acid, 3 % glyoxal/0.8 % acetic acid/20 % ethanol, or 9 % glyoxal/8 % acetic acid, suggesting that acetic acid impairs ICD immunostaining (Supp. Fig. 1B, right panel). Conversely, fixation with 3 % glyoxal or 3 % glyoxal/20 % ethanol showed a much more restricted ICD staining pattern, importantly, providing a clear distinction between N-Cadherin and Cx43, and allowing for better visualization of their spatial separation (Fig. 3A, trace analysis panel). This finding is consistent with previous reports that gap junctions occupy the most apical position of the ICD, while adherens junctions are located at the adhesion belt [8,11,12]. Overall, our study demonstrates that glyoxal improves ICD immunostaining in mouse hearts, while the inclusion of acetic acid in the fixative should be avoided.

### 3.4 Evaluating glyoxal's impact on sarcomere immunostaining

**Fig. 4:**
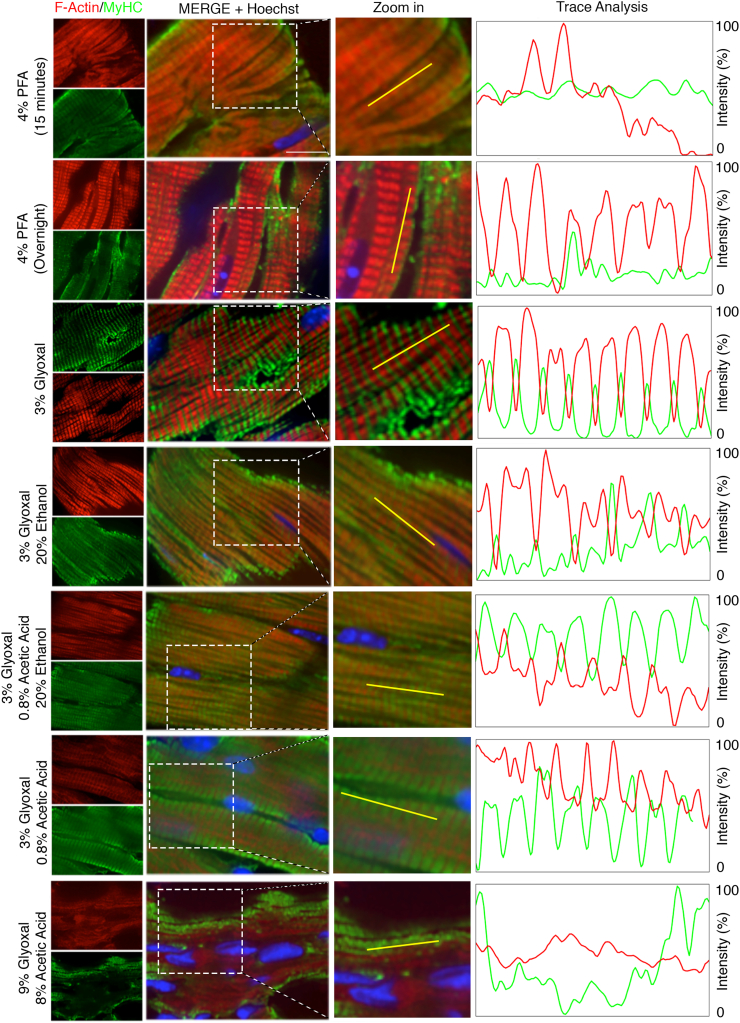
Immunofluorescence of filamentous actin (F-Actin) and myosin heavy chain (MyHC) in mouse hearts. Representative immunofluorescent images of sarcomeric proteins MyHC (green) and F-actin (red) in murine cardiac tissues with proposed fixatives. Scale bar = 20 μm. Right panels, trace analyses of MyHC and F-actin signals in tissues with fixatives. Of all samples, MyHC and F-actin signals had the best 180 °C phase shift in the heart fixed with 3 % glyoxal, followed by 3 % glyoxal/0.8 % acetic acid and 3 % glyoxal/0.8 % acetic acid/20 % ethanol. Gly, glyoxal. EtOH, ethanol. AA, acetic acid. (For interpretation of the references to colour in this figure legend, the reader is referred to the web version of this article.)

**Supplementary Fig. 2:**
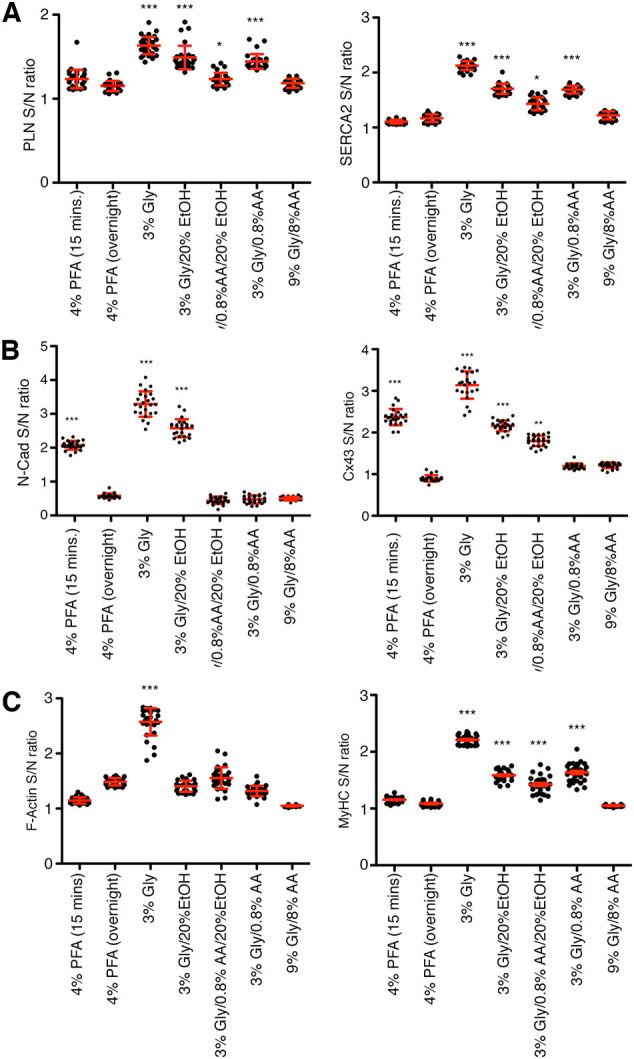
Unbound antibodies and small molecules were effectively removed in 3 % glyoxal-fixed mouse hearts. Immunostaining showing that Alex488-conjugated secondary antibody and Alexa586-conjugated phalloidin were retained in mouse cardiac sections fixed with 4 % PFA. However, the signals were improved in 3 % glyoxal fixative. Scale bar = 20 μm.

Another protein-rich region of cardiomyocytes is the sarcomere. We sought to determine whether glyoxal would similarly enhance antigenicity in this region, as observed with SR proteins. For this analysis, we stained filamentous actin (F-actin) using red fluorochrome-conjugated phalloidin (molecular weight, 1300 Da) and myosin with an antibody (150 kDa). Similar to PLN staining, myosin antibodies produced positive staining at the PM in 4 % PFA-fixed tissues (Fig. 4A). While sarcomere staining appeared intact in these samples, it exhibited a reduced signal-to-noise ratio (Supp. Fig. 1C) 1.48 ± 0.01 F-actin, 1.09 ± 0.01 MyHC for overnight 4 % PFA fixation). Actinomyosin staining in glyoxal-fixed tissues (excluding 9 % glyoxal/8 % acetic acid) was significantly improved and exhibited higher signal-to-noise ratios compared to 4 % PFA-fixed tissues (Fig. 4B, 2.21 ± 0.02 MyHC for 3 % glyoxal, *p* < 0.001; 1.60 ± 0.02 for 3 % glyoxal/20 % ethanol, p < 0.001; 1.42 ± 0.03 for 3 % glyoxal/20 % ethanol/0.8 acetic acid, p < 0.001; 1.64 ± 0.03 for 3 % glyoxal/0.8 % acetic acid, p < 0.001). Among these, 3 % glyoxal yielded the most pronounced sarcomere staining with the highest signal-to-noise ratio (2.58 ± 0.05 actin, 2.21 ± 0.02 MyHC for 3 % glyoxal, p < 0.001 when compared to overnight 4 % PFA-fixed hearts). We also assessed the quality of sarcomere fluorescent images with an analysis software SOTA [13], which provides a score relative to sarcomere organization. Overnight fixation with 4 % PFA and 3 % glyoxal resulted in the highest F-actin sarcomere organization score, while 9 % glyoxal/8 % acetic acid showed the lowest F-actin score (0.022 ± 0.002 for 15 min 4 % PFA, 0.220 ± 0.008 for overnight 4 % PFA, 0.227 ± 0.014 for 3 % glyoxal, 0.163 ± 0.015 for 3 % glyoxal/20 % ethanol, 0.156 ± 0.012 for 3 % glyoxal/0.8 % acetic acid/20 % ethanol, 0.027 ± 0.002 for 3 % glyoxal/0.08 % acetic acid, and 0.006 ± 0.001 for 9 % glyoxal/8 % acetic acid). In parallel, 3 % glyoxal also had the highest sarcomere organization score among all fixative solutions when assessing MyHC staining images (0.053 ± 0.009 for 15 min 4 % PFA, 0.063 ± 0.016 for overnight 4 % PFA, 0.169 ± 0.016 for 3 % glyoxal, 0.072 ± 0.008 for 3 % glyoxal/20 % ethanol, 0.069 ± 0.005 for 3 % glyoxal/0.08 % acetic acid/20 % ethanol, 0.057 ± 0.005 for 3 % glyoxal/0.08 % acetic acid, 0.009 ± 0.003 for 9 % glyoxal/8 % acetic acid). Consistent with previous SR and ICD findings, fixation with 9 % glyoxal/8 % acetic acid abolished sarcomere staining (Figs. 4A and Supp. Fig. 1C), suggesting that high glyoxal concentrations disrupt actinomyosin structure, or more likely, reduced the actinomyosin-antibody binding. Immunostaining controls were examined to validate staining specificity. Supplementary Fig. 2 shows that staining with Alexa488-conjugated secondary antibodies alone lightly marked the extracellular region in 3 % glyoxal-fixed tissues, while the signals permeated cardiomyocytes (counter-stained with phalloidin) in 4 % PFA-fixed tissues, indicating that secondary antibodies were more easily trapped in 4 % PFA-fixed tissues. In keeping with our findings in Fig. 4, phalloidin staining was also improved in 3 % glyoxal-fixed tissues compared to 4 % PFA. Together, these results demonstrate that 3 % glyoxal is an effective fixative for sarcomere visualization, enhancing antibody-antigen interaction and reducing unbound antibodies.

### 3.5 Evaluating glyoxal fixation on antibody penetration in cardiac tissues

**Fig. 5:**
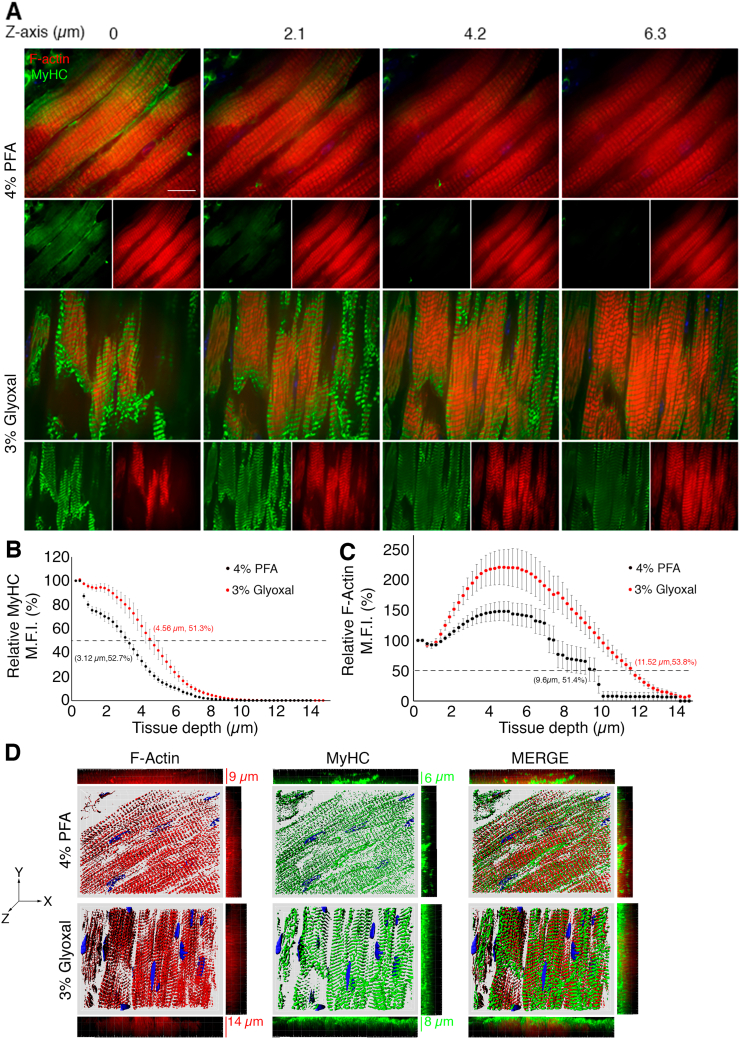
3 % Glyoxal enabled antibody penetration in deeper cardiac sections. (A) Representative images of stepwise z-axis acquisition of fixed cardiac sections stained with MyHC antibody and phalloidin. MyHC signals faded at 2.1 μm of 4 % PFA-fixed sections, but remained visible in 6.3 μm of 3 % glyoxal-fixed sections. Actin signals were visible across all layers of tested sections irreversible of fixatives. Scale bar = 20 μm. (B—C) Dot plots showed that MyHC and F-actin signals were better preserved qualitatively in cardiac sections fixed with 3 % glyoxal than 4 % PFA-fixed tissues. The tissue depth where 50 % signal intensity remained was indicated and highlighted. *n* > 18 images at 0.24-μm depth from 3 independent cardiac tissues. All dot plots in the figure represent mean ± SEM. Welch's *t*-test post-hoc analysis was performed to assess the statistical significance. *, *p* < 0.05. (D) Representative topographical images of Imaris-assisted F-Actin and MyHC from both 4 % PFA- or 3 % glyoxal-fixed cardiac sections. Both XZ and YZ orthogonal images were also included for assessing the quality of deeper cardiac staining. Both F-actin and MyHC staining were less uniform (spotty) in 4 % PFA-fixed sections, but were homogenous in 3 % glyoxal-fixed sections.

A common issue with formaldehyde-based fixatives is over-crosslinking of macromolecules, which can render epitopes inaccessible and restrict antibody movement. Our results (Fig. 2, Fig. 3, Fig. 4 and Supp. Fig. 1) and previous studies [3,4] suggest that reduced crosslinks by glyoxal in tissues may enable deeper antibody penetration and enabling more effective removal of unbound antibodies. To test this hypothesis, we repeated F-actin and MyHC immunofluorescence in 30-μm thick cardiac sections. As shown in Fig. 5A, phalloidin-based F-actin staining remained consistent across 6 μm of 4 % PFA-fixed tissues, while MyHC staining gradually diminished with increasing tissue depth (z-axis). In contrast, MyHC staining in 3 % glyoxal-fixed tissues was unaffected, maintaining intact sarcomere patterns throughout this imaging depth. These findings were confirmed by measurement of mean fluorescent intensity (M.F.I.) for both MyHC and F-actin (Fig. 5B and C), which showed greater signal preservation in glyoxal-fixed tissues at deeper layers (e.g., 51.3 % MyHC signal remained at 4.56 μm depth in 3 % glyoxal tissues vs. 52.7 % at 3.12 μm depth in 4 % PFA tissues). Notably, the F-actin MFI signals increased between 2- and 6-μm depths and gradually decreased after 6-μm in both 4 % PFA and 3 % glyoxal-fixed tissues (Fig. 5C). However, this pattern was not observed for MyHC staining (Fig. 5B), suggesting that small molecules like phalloidin can easily penetrate tissues and settle between 2- and 10-μm tissue depth, whereas the movement of larger molecules, such as MyHC antibody, is still restricted in comparison. We also assessed immunostaining quality using computer-assisted reconstruction of z-stack images and orthogonal plane views (xz and yz, Movie 1, Movie 2). MyHC signals in 4 % PFA-fixed tissues appeared disorganized, while specimens fixed in 3 % glyoxal showed well-aligned sarcomeres. Importantly, analyses of orthogonal plane images revealed ‘sporadic’ staining pockets in 4 % PFA-fixed tissues, while the staining appeared much more uniform and consistent in tissues fixed with 3 % glyoxal (Fig. 5D). The sporadic F-actin and MyHC staining is likely due to excessive crosslinks in 4 % PFA treated tissues, which may cause fluorochromes to become trapped. Taken together, these results demonstrate that 3 % glyoxal fixation enhances antibody penetration, preserves antigenicity, and improves the quality of immunostaining across greater tissue depths in cardiac sections (Supp. Fig. 1).

### 3.6 Evaluating glyoxal fixation on cardiac resident cells immunostaining

**Fig. 6:**
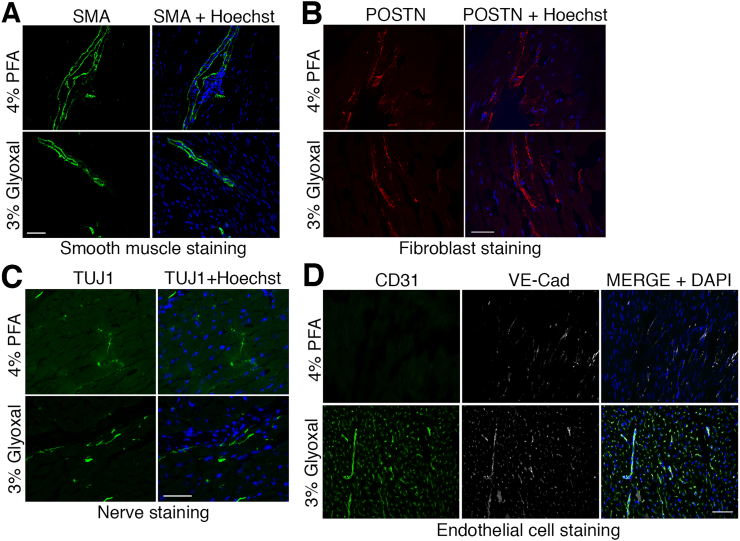
Glyoxal improved the visualization of cardiac resident cells in mouse hearts. (A) Representative fluorescent microscopy of smooth muscle actin (SMA) in mouse hearts. SMA staining of arteries in 4 % PFA-fixed cardiac sections appeared only on both sides of smooth muscle layers, but was more complete in 3 % glyoxal-fixed sections. (B) Representative fluorescent microscopy of fibroblasts via periostin (POSTN) staining in mouse hearts. (C) Representative images of nerve systems in mouse hearts via β-Tubulin III (TUJ1) immunostaining. An increased background noise was detected in 4 % PFA-fixed sections, leading to reduced TUJ1 signals. The use of 3 % glyoxal qualitatively improved TUJ1 staining with a reduced background noise. (D) Representative fluorescent microscopy of endothelial cells in mouse hearts. Endothelial cells were visualized via CD31 and VE-Cadherin (VE-Cad) immunostainings. CD31 signal (green) was absent in 4 % PFA-fixed cardiac tissues but availed in 3 % glyoxal-fixed sections. This was reaffirmed by an increased VE-Cad signal (grey). Scale bar = 50 μm. (For interpretation of the references to colour in this figure legend, the reader is referred to the web version of this article.)

Our results demonstrate that glyoxal, at low concentration, is advantageous for cardiomyocyte immunostaining in mouse cardiac tissues (Fig. 2, Fig. 3, Fig. 4, Fig. 5), but its effect on cardiac resident cells remains unassessed. To address this, we stained smooth muscle cells, fibroblasts, and endothelial cells with cell-type specific antigens in 4 % PFA- and 3 % glyoxal-fixed tissues. Immunostaining with a smooth muscle actin (SMA) antibody highlighted 2 thin layers of smooth muscle cells juxtaposed to internal/external elastic lamina in 4 % PFA-fixed tissues but captured the entirety of smooth muscle cells in 3 % glyoxal tissues (Fig. 6A). The finding was reaffirmed by a higher, but insignificant, M.F.I. signal in 3 % glyoxal-fixed tissues than 4 % PFA (100 ± 18.96 % for 4 % PFA, 149.30 ± 14.41 % for 3 % glyoxal, *p* = 0.065). Periostin (POSTN) staining for cardiac fibroblasts appeared indifferent qualitatively and quantitatively between the 2 fixatives (Fig. 6B). The nervous system in cardiac tissues was visualized by β-Tubulin III (TUJ1) immunostaining, which showed a 7-fold increase in TUJ1 signals in 3 % glyoxal-fixed tissues in comparison to 4 % PFA-fixed tissues (787.993 ± 45.711 % in 3 % glyoxal vs. 100 ± 18.50 % in 4 % PFA, *p* < 0.001) (Fig. 6C). This may result from an improved signal-to-noise ratio in IF. Resident endothelial cells were visualized by CD31 and VE-Cadherin (VE-Cad) co-staining. CD31 signals showed a significant 12 % improvement in 3 % glyoxal-fixed tissues compared to 4 % PFA-fixed tissues (100 ± 2.66 % in 4 % PFA; 112.62 ± 4.29 % in 3 % glyoxal, *p* < 0.05), while VE-Cad signals were comparable in both conditions (Fig. 6D). Together, these results suggest that glyoxal can be a superior fixative for staining some cardiac resident cells and does not negatively impact immunostaining for others.

### 3.7 Evaluating glyoxal fixation on protein solubility

**Fig. 7:**
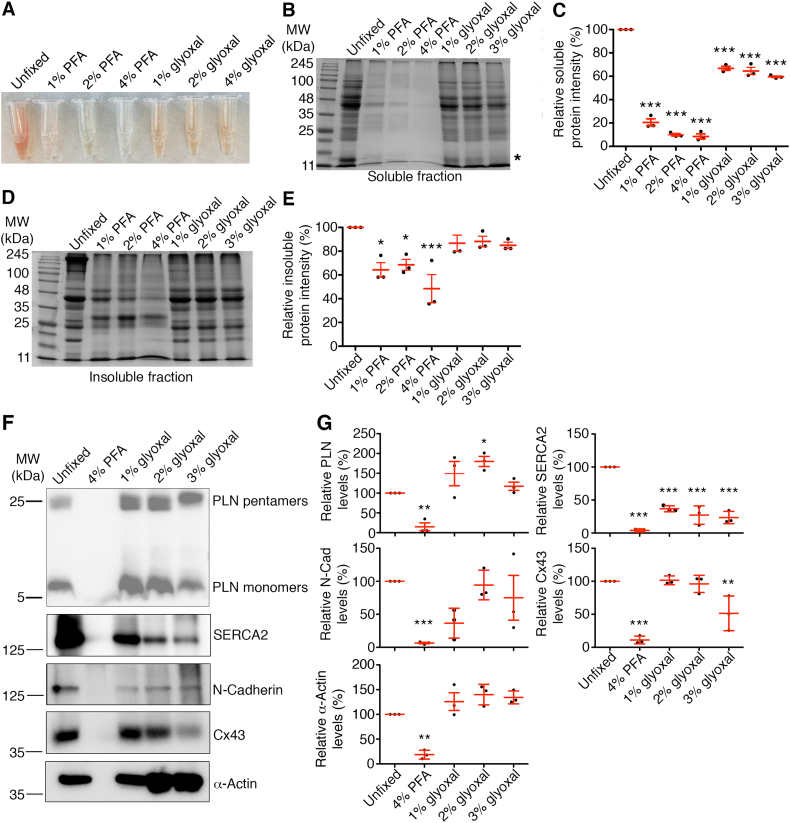
Glyoxal unevenly crosslinked proteins in mouse hearts. (A) Cell-free, soluble protein lysates extracted from mouse hearts following an overnight chemical crosslink and centrifugation. (B) A representative image of Coomassie brilliant blue-stained SDS-PAGE gel for soluble protein lysates. (C) Quantification of soluble protein intensity of SDS-PAGE/Coomassie staining (*n* = 3). (D) A representative image of Coomassie brilliant blue-stained SDS-PAGE gel for insoluble protein lysates. (E) Quantification of insoluble protein intensity of SDS-PAGE/Coomassie staining (n = 3). (F) Immunoblots of PLN, SERCA2, N-Cadherin (N-Cad), Cx43, and α-Actin extracted from the mouse hearts following overnight fixation with 4 % PFA, 1 % glyoxal, 2 % glyoxal, or 3 % glyoxal. (G) Quantification of selected proteins by immunoblots in (F). n = 3 independent hearts. All dot plots in the figure represent mean ± SEM. Welch's *t*-test post-hoc analysis was performed to assess the statistical significance. *, p < 0.05; **, *p* < 0.01; ***, p < 0.001. (For interpretation of the references to colour in this figure legend, the reader is referred to the web version of this article.)

Given the superior immunostaining patterns and enhanced antibody penetration observed in cardiac sections, we next aimed to characterize the fixative properties of glyoxal in cardiac tissue. We extracted both soluble (with RIPA buffer) and insoluble protein (with 8 M urea) lysates from mouse hearts fixed overnight in 1 % PFA, 2 % PFA, 4 % PFA, 1 % glyoxal, 2 % glyoxal, and 3 % glyoxal. Fresh cardiac tissues, snap frozen in liquid nitrogen, were included as a negative control (unfixed). Fig. 7A illustrates the varying effects of fixatives on tested cardiac proteins. The soluble protein lysates extracted from unfixed hearts exhibited the strongest orange hue, while lysates from PFA-fixed hearts were readily translucent. Furthermore, in glyoxal-fixed lysates, an inverse relationship was observed between hue intensity and glyoxal concentration, with higher concentrations resulting in less-intense hues. These results suggests that glyoxal is a less potent fixative than 4 % PFA, allowing more proteins to remain soluble after glyoxal fixation. We then performed polyacrylamide gel electrophoresis (SDS-PAGE) and visualized in-gel proteins using Coomassie blue staining. Fig. 7B confirms that PFA and glyoxal derivatives differentially affect cardiac protein solubility. Quantitative analysis indicated that approximately 8–20 % of proteins remained soluble in PFA-fixed samples compared to unfixed cardiac lysates (Fig. 7C, 20.49 ± 3.03 % for 1 % PFA, *p* < 0.001; 9.90 ± 1.04 % for 2 % PFA, p < 0.001; 8.81 ± 2.00 % for 4 % PFA, p < 0.001). In comparison, at least 50 % of proteins remained soluble in glyoxal-fixed samples, with increasing glyoxal concentration, resulting in a gradual reduction of soluble proteins: 66.83 ± 1.72 % for 1 % glyoxal (*p* < 0.05), 64.56 ± 3.09 % for 2 % glyoxal (p < 0.05), and 59.60 ± 0.83 % for 3 % glyoxal (p < 0.05). For example, Coomassie gel examination revealed a protein band at 17 kDa, which diminished in intensity with increasing glyoxal concentration (Fig. 7B, asterisk). This protein likely corresponds to myoglobin, which could explain the gradual decrease in orange hue observed in the protein lysates (Fig. 7A). Next, we employed immunoblots to determine how glyoxal affects the cardiac proteins investigated in our IF studies. Fig. 7C and D demonstrate that the solubility of sarcoplasmic reticulum proteins SERCA2 and PLN varied significantly. PLN protein levels were elevated in all glyoxal-fixed tissue lysates compared to unfixed controls, though statistical significance was only achieved at the 2 % concentration. Interestingly, SERCA2 levels decreased significantly across all glyoxal-fixed tissue lysates compared to unfixed controls. The ICD protein N-Cadherin showed an initial decrease at 1 % glyoxal but remained stable with higher glyoxal concentrations. In contrast, the other ICD protein Cx43 gradually decreased as glyoxal concentration rose to 3 %. α-actin levels increased at 2 % and 3 % glyoxal while remaining unchanged at 1 % glyoxal. These results indicate that glyoxal fixation affects the solubility of cardiac proteins differentially, leading to a relative enrichment of some proteins in fixed tissue lysates.

**Fig. 8:**
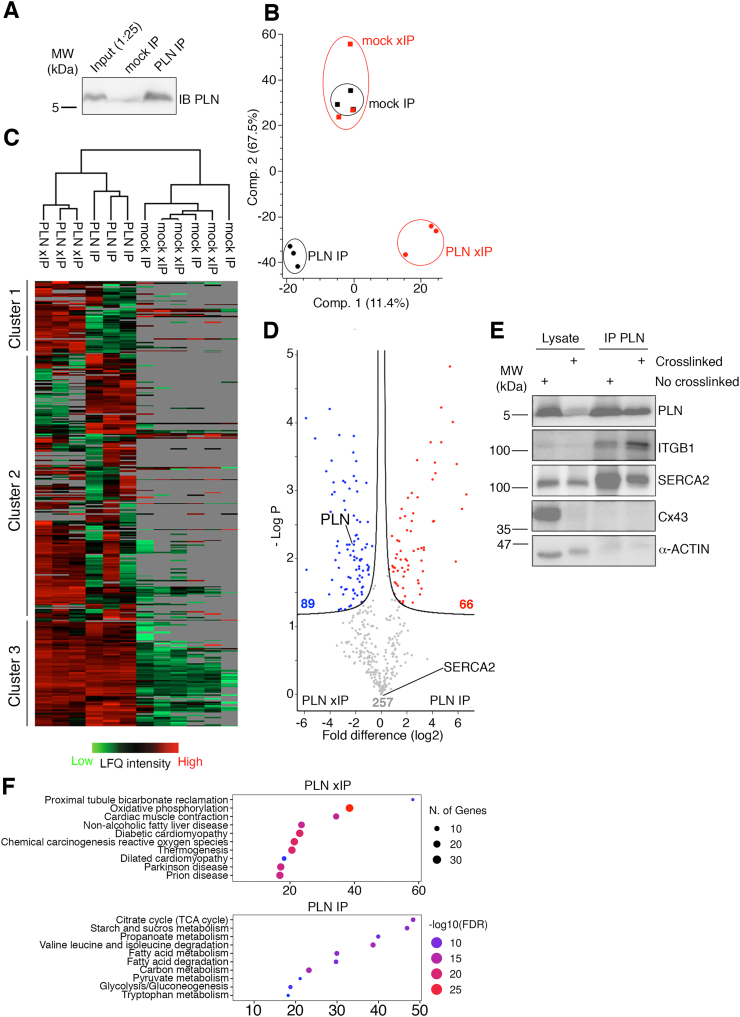
Glyoxal improved IP-MS outcomes with mouse cardiac tissues. (A) A representative immunoblot of PLN immunoprecipitation from mouse cardiac protein lysates. (B) PCA analysis demonstrated that PLN interactomes in mouse hearts were affected by glyoxal. (C) Unsupervised heatmap analysis showed that co-fractionated PLN-interacting proteins clustered into 3 different groups. Proteins in cluster 1 were enriched in 3 % glyoxal compared to unfixed tissues, while cluster 2 contained proteins whose enrichment varies between unfixed and fixed tissues. (D) Volcano plot highlighted proteins that were statistically enriched in IP (red dots, 66 proteins) or xIP (blue dots, 89 proteins). (E) Co-immunoprecipitation assays validating PLN xIP-MS results in Figs. (B—D). The chemiluminescent signals of co-precipitated ITGB1 were stronger in xIP than IP groups, while SERCA2 signals were comparable between xIP and IP groups. (F) GO/KEGG analyses highlighted the biological functions of the protein groups identify in IP (below) or xIP groups (above). Analysis was performed with ShinyGO at 0.05 FDR cut-off. The top 10 functional groups of each sample set were listed. (For interpretation of the references to colour in this figure legend, the reader is referred to the web version of this article.)

### 3.8 Evaluating glyoxal's applicability for xIP-MS

Formaldehyde-based xIP-MS has been proven to be an effective method for studying protein complexes in cells [14,15], but its excessive crosslinking can lead to insoluble protein complexes that complicate mass spectrometry analysis. We hypothesized that the mild crosslinking property of glyoxal makes it an effective alternative to formaldehyde by improving protein solubility and downstream protein identification by mass spectrometry. Here, we performed immunoprecipitation assays to enrich PLN and associated proteins in unfixed (IP) or 3 % glyoxal-fixed (xIP) mouse tissues (Fig. 8A), followed by protein identification using mass spectrometry. We have had a longstanding research focus on PLN and its interactome [9,[16], [17], [18], [19]] and chose this protein to explore the xIP methodology with glyoxal. xIP with 4 % PFA was not included, since no soluble PLN was detected (Fig. 7F). In total, 412 unique proteins were recovered. Principal component analysis (PCA) showed that mock immunoprecipitation (without primary antibody) clustered together, irrespective of fixation condition, indicating similar levels of background non-specific protein enrichment (Fig. 8B, black and red squares). Contrarily, IP (red circles) and xIP groups (black circles) were clearly separated into 2 clusters. The effectiveness of glyoxal fixation was reaffirmed by the unsupervised clustering heatmap, which divided 412 proteins into 3 distinctive groups (Fig. 8C). Cluster 1 contained enriched proteins in xIP samples, cluster 2 contained proteins predominantly enriched in IP samples, and cluster 3 contained proteins that were enriched in both IP and xIP groups, but not in mock immunoprecipitation experiments. Volcano plot showed that 89 and 66 proteins were significantly enriched (FDR < 0.05) in xIP (blue circles) and IP samples (red circles), respectively (Fig. 8D). Some significantly enriched proteins in xIP groups include desmoplakin, integrin beta-1 (ITGB1), Na^+^/K^+^ transporting ATPase, and phospholemman (Supplemental Data, Table 1), whose functional interactions with PLN have been reported [[20], [21], [22]]. In comparison, the proteins that were significantly enriched in IP groups had mitochondrial origins (Supplemental Data, Table 2), which is consistent with previous findings [23]. Interestingly, PLN was enriched by 4-fold following glyoxal fixation, while its interacting proteins - including SERCA2 [24], 14–3-3 [25], poly-ubiquitin B [19] - were equally enriched in both IP and xIP groups, suggesting that glyoxal preserves the detection of known PLN-protein interaction in intact hearts. For validation, we performed co-immunoprecipitation assays with unfixed or glyoxal-fixed cardiac tissues. In agreement with our mass spectrometry results, signals of co-precipitated ITGB1 were significantly higher in xIP than IP groups (100 ± 21.63 % for IP vs. 281.95 ± 33.20 % for xIP; *p* = 0.01) (Fig. 8E). In comparison, SERCA2 signals were comparable between both groups (131.90 ± 22.57 % for IP vs. 100 ± 12.88 % for xIP). Negative controls, including Cx43 and α-actin, were not co-precipitated with PLN antibodies, demonstrating that the co-IP assays were stringent. It is noteworthy that some α-actin proteins slightly shifted upward on the blot, likely due to crosslinks. Lastly, we aimed to elucidate the functional importance of the proteins enriched in unfixed and fixed tissues using Gene Ontology (GO) and Kyoto Encyclopedia of Gene and Genomes (KEGG). Enriched proteins from both IP and xIP groups were analyzed with ShinyGO [10] using the *Mus musculus* database and an FDR < 0.05 cut-off. Our results showed that functional pathways related to cardiac muscle contraction, diabetic cardiomyopathy, and dilated cardiomyopathy were enriched in xIP groups, while metabolic pathways involving fatty acids, proteins, and glucose were highlighted in the IP groups (Fig. 8E). These results indicate that glyoxal-assisted IPMS is perhaps more effective at capturing PLN-associated proteins functionally implicated in cardiomyopathy and that glyoxal xIP-MS may provide additional or refined information that would have been missed in unfixed or PFA-fixed tissues.

## 4 Discussion

Formaldehyde, typically used as 10 % formalin or 4 % paraformaldehyde (PFA), has historically served as the standard fixative in pathological investigations; however, formaldehyde-based fixation presents several technical challenges in modern applications. Like alcohol-based fixatives, formaldehyde exposure leads to specimen shrinkage and distortion [26]. Additionally, covalent methylene bridges formed by formaldehyde can disrupt antigen-antibody interactions [27], often generating staining artifacts in IF [28]. Although techniques such as antigen retrieval and protease digestion have been used to mitigate these issues [3], these methods often yield inconsistent results and can introduce additional complications in downstream analyses. Considering these concerns, glyoxal has emerged as a promising alternative to formaldehyde for several reasons. First, glyoxal is commercially available as a 40 % (*w*/*v*) solution, which can be diluted for immediate use, eliminating the need for the hazardous preparation procedures associated with formaldehyde-based fixatives. Furthermore, a previous study demonstrated that glyoxal is non-volatile in its aqueous phase [29], making it a safer option to formaldehyde, which releases toxic and carcinogenic formaldehyde gas. Second, glyoxal has been shown as an effective and fast fixative across different cell types and mouse brain tissues [3,4]. Future studies in other mouse and human non-cardiac tissues examining imaging such as confocal-based imaging and immunohistochemistry, along with various downstream biochemical applications, such as immunoprecipitation and immunoblotting, will ultimately need to be performed to determine glyoxal's broad based utility. Lastly, glyoxal fixation is less disruptive to antigen-antibody binding, enhancing antigenicity in cells [4], murine brain tissues [3], zebrafish embryos [6], Drosophila embryos [5], and human biopsies [30]. Additionally, several studies have reported that glyoxal as a fixative has significantly improved downstream biological applications, such as RNA fluorescent in situ hybridization [5,31], RNA extraction for transcriptomic analyses [32,33], and does not impair electron microscopy [3]. Our findings support these studies, showing that, among various glyoxal concentrations tested, 3 % glyoxal provides superior fixation for cardiac tissues when compared to 4 % PFA.

Our study evaluated 5 glyoxal solutions, including varying concentrations with and without additives such as acetic acid and ethanol (Table 1). While acetic acid facilitates glyoxal diffusion in cells [4], ethanol could prevent protein aggregation [34] and provide additional fixation [3]. Our results indicate that low-concentration glyoxal fixatives (3 %) effectively preserve myofiber structure, while higher concentrations (9 %) lead to significant myofiber shrinkage. This contrasts with a previous study that found high concentrations of glyoxal (9 % glyoxal/8 % acetic acid) improved tissue solidity in murine brain tissues [3]. In cardiac tissues, however, 9 % glyoxal/8 % acetic acid caused over-crosslinking, tissue brittleness (Fig. 1, Fig. 2, Fig. 3, Fig. 4), and increased fragility during cryosectioning, where the tissues were more likely to detach from the OCT medium. We also tested glyoxal mixtures with acetic acid and/or ethanol, which have different utilities. While acidified glyoxal fixatives (pH 4–5) with acetic acid evidently improve immunofluorescent signals in cells [4], Kanno et al. reported that a high concentration (8 %) of acetic acid dehydrates mouse brain tissues, further improving soft tissue preservation [3]. Ethanol improves glyoxal-based fixatives via an enhanced dehydration and alcohol-mediate crosslinks [4]. Although these studies support the inclusion of acetic acid and alcohol in glyoxal fixatives for cell and mouse brain tissue preservation, our investigation demonstrates that these additives had minimal effect on cardiac immunostaining when compared to 3 % glyoxal alone. In our studies, the addition of acetic acid or ethanol to the 3 % glyoxal solution impaired immunostaining of cardiomyocyte organelle structures, suggesting that these additives interfere with cardiac tissue preservation (Fig. 1, Fig. 2, Fig. 3, Fig. 4). These findings underscore the importance of optimizing glyoxal fixation conditions (e.g., concentration, additives, pH) to tissue type. Specifically for cardiac tissues, our results support the use of 3 % glyoxal for IF and tissue integrity.

Our findings demonstrate that 3 % glyoxal offers several distinct advantages over conventional 4 % PFA fixation for IF in murine cardiac tissues. First, 3 % glyoxal significantly improved the signal-to-noise ratio for sarcoplasmic reticulum, intercalated disc, and sarcomeric proteins (Fig. 2, Fig. 3, Fig. 4, Fig. 5). Improvements were also observed for certain cardiac resident cells (Fig. 6). These enhancements are likely due to reduced inter- and intracellular covalent crosslinking with glyoxal, which facilitates greater antigen-antibody interactions, enhances antibody penetration, and improves the removal of unbound fluorochromes during washing procedures. This is particularly evident in the uniform distribution of F-actin and MyHC signals in 3 % glyoxal-fixed tissues, in contrast to the sporadic and hazy signals observed with 4 % PFA fixation (Fig. 5D, xz- and yz-planes). Second, 3 % glyoxal minimized staining artifacts. We observed exaggerated fluorescent signals at the plasma membrane (PM) in PFA-fixed tissues, which were absent in glyoxal-fixed samples (Fig. 2, Fig. 4). This PM artifact, commonly reported in murine cardiomyocytes [9,35], iPSC-derived cardiomyocytes [36], and human cardiac biopsies [37], may be more widespread than previously appreciated. While the cause of the PFA-induced PM artifact remains unclear, our results suggest that glyoxal effectively eliminates this issue. Lastly, glyoxal is compatible with common surfactants, including Triton X-100, Tween-20, and methanol, used for PM permeabilization in immunofluorescence protocols. This compatibility allows for seamless integration of glyoxal into existing workflows with minimal procedural adjustments.

To assess the crosslinking strength of glyoxal, we performed SDS-PAGE on soluble proteins extracted from mouse hearts, comparing unfixed tissues with those fixed using 1 %, 2 %, and 4 % PFA, and 1 %, 2 %, and 3 % glyoxal. Following Coomassie blue staining (Figs. 7B & 7D), we found that glyoxal lightly crosslinked proteins, with 60 % of cardiac proteins remaining soluble. In contrast, PFA fixation caused a loss of over 80 % of soluble proteins due to excessive crosslinking. The lightly crosslinked proteins in glyoxal-fixed tissues were still amenable to downstream biochemical analyses, including immunoblotting and crosslinking immunoprecipitation-mass spectrometry (xIP-MS) (Fig. 7, Fig. 8). This characteristic is particularly advantageous when working with rare or limited human biopsies that require multiple experiments. However, we observed that glyoxal differentially crosslinked cardiac proteins (Fig. 7F). Previous studies report that glyoxal-mediated crosslinks occur via the ε amines of l-lysine [38,39], and post-translational modifications on these amino acids may interfere glyoxal fixation. Similarly, proteins with enriched lysine/arginine peptidyl stretches (e.g., nuclear localization signal [40]) may be preferentially targeted, resulting in an impaired epitope recognition or reduced protein solubility in downstream biochemical applications. These limitations should be considered when applying glyoxal. Overall, our results demonstrate that low concentrations of glyoxal effectively preserve cardiac tissues without excessive crosslinking, are compatible with common detergents, and enable both biochemical and histological analyses.

Tissue xIP-MS holds significant promise for studying protein interactomes and biochemical changes in pathological samples. However, the use of formaldehyde as a fixative in xIP-MS presents 2 key challenges. First, formaldehyde promotes protein aggregation, even at low concentrations (0.1 % *w*/*v*%), leading to the elimination of these aggregated proteins from the soluble fraction prior to affinity purification [34]. In agreement with this, we found that approximately 80 % of soluble proteins were lost when mouse hearts were fixed in 1 % PFA overnight (Fig. 7A and C). The second challenge is that formaldehyde crosslinking impairs mass spectrometry searches. The random deposition of methylene bridges between proteins adds 12 or 24 kDa to affected peptides, altering their mass-to-charge ratios and interfering with protein detection by mass spectrometry [28,41,42]. In this study, we explored glyoxal's potential for tissue xIP-MS due to its faster fixation [4] and reduced crosslinking compared to traditional PFA (Fig. 7). Our results demonstrated several advantages of glyoxal-based xIP-MS. First, glyoxal efficiently preserved protein complexes in tissues, as shown by PCA analysis, which clearly differentiated IP (black circles) and xIP (red circles) samples (Fig. 8B). Second, glyoxal crosslinking did not negatively affect protein interactions, including that of phospholamban (PLN) with SERCA2 [24], 14–3-3 [25], and poly-ubiquitin B [19]. This was confirmed by equal enrichment of these proteins in both IP and xIP groups (Supplemental Data, Table 3). Third, glyoxal preserved macromolecules in tissues, as evidenced by a 4-fold enrichment of PLN in xIP samples compared to unfixed controls (Fig. 8D, Supplemental Data, Table 1). We also identified integrin beta-1 and phospholemman as PLN-interacting proteins exclusively in the xIP groups. Although functional interactions of these proteins with PLN have been reported elsewhere [[20], [21], [22]], our data suggest that PLN homopentamers favour protein-protein interactions. It is also possible that the native PLN-integrin beta-1 (or PLN-phospholemman) interaction is transient and could only be detected when proteins are crosslinked. It is noteworthy that other proteins previously reported to interact with PLN, such as HS-associated protein X-1 (HAX-1) [43,44], protein kinase A (PKA) [24], and calmodulin-dependent protein kinase II (CaMKII) [45], von Hippel-Lindau [46], were not detected in our mass spectrometry analysis. These proteins are involved in PLN's post-translational modifications, with their interactions typically induced by specific signaling pathways (e.g., PKA activation) or stress conditions (e.g., HAX-1 and CaMKII). The absence of these interactions in our study may be attributed to the use of physiological heart conditions in the mass spectrometry experiments, where the requisite signaling events or stress responses necessary for their activation were not present, potentially preventing their detection. Overall, our findings show that glyoxal lightly crosslinks proteins, preserving complex structures while maintaining protein solubility for downstream biochemical applications such as affinity purifications (Fig. 7). Moreover, glyoxal fixation does not interfere with mass spectrometry analysis, in contrast to formaldehyde. To our knowledge, we are the first group to perform tissue xIP-MS on murine cardiac tissues. Although our findings support the use of glyoxal for tissue xIP-MS as a proof-of-principle study, its efficacy will need to be further determined across different tissues and proteins-of-interest.

The following are the supplementary data related to this article.

Supplementary material Excel data file containing all mass spectrometry derived data. Shown are protein IDs, gene names, protein descriptions, fold-difference, and -Log *P* values. Table 1: PLN-associated proteins significantly enriched in only glyoxal-fixed hearts. Table 2: PLN-associated proteins significantly enriched in only unfixed hearts. Table 3: PLN-associated proteins commonly identified in both the glyoxal-fixed and unfixed hearts. Supplementary material

Movie 1 Imaris-assisted topography of F-actin and MyHC immunostaining in 4 % PFA-fixed cardiac section. Movie 1

Movie 2 Imaris-assisted topography of F-actin and MyHC immunostaining in 3 % glyoxal-fixed cardiac section. Movie 2

Supplementary material Image 1

## CRediT authorship contribution statement

**Allen C.T. Teng:** Writing – original draft, Visualization, Validation, Project administration, Methodology, Investigation, Formal analysis, Conceptualization. **Dev Mehangrey:** Methodology, Funding acquisition. **Ava Vandenbelt:** Writing – review & editing, Writing – original draft, Methodology, Formal analysis. **Karl Vearncombe:** Methodology, Formal analysis. **Justin D. Callahan:** Writing – review & editing, Methodology, Formal analysis. **Priya Mistry:** Writing – review & editing, Methodology, Formal analysis. **Wenping Li:** Writing – review & editing, Project administration, Methodology, Formal analysis. **Cristine J. Reitz:** Writing – review & editing, Methodology. **Omar Hamed:** Writing – review & editing, Methodology. **Madison Roche:** Writing – review & editing, Methodology, Formal analysis. **Uros Kuzmanov:** Writing – review & editing, Methodology, Formal analysis, Data curation. **Jason E. Fish:** Writing – review & editing, Supervision, Resources. **Slava Epelman:** Writing – review & editing, Supervision, Resources. **Anthony O. Gramolini:** Writing – review & editing, Writing – original draft, Supervision, Resources, Project administration, Methodology, Funding acquisition, Conceptualization.

## Declaration of competing interest

The authors declare that they have no known competing financial interests or personal relationships that could have appeared to influence the work reported in this paper.
