# Supplementary material for: Glyoxal is a superior fixative to formaldehyde in promoting antigenicity and structural integrity in murine cardiac tissues
Source: J Mol Cell Cardiol Plus. 2025 May 11;12:100454. doi: 10.1016/j.jmccpl.2025.100454 (PMC12145851; doi:10.1016/j.jmccpl.2025.100454)
Supplement: Supplementary file 4 — Supplementary material [file mmc6.pdf]

Figures 7B

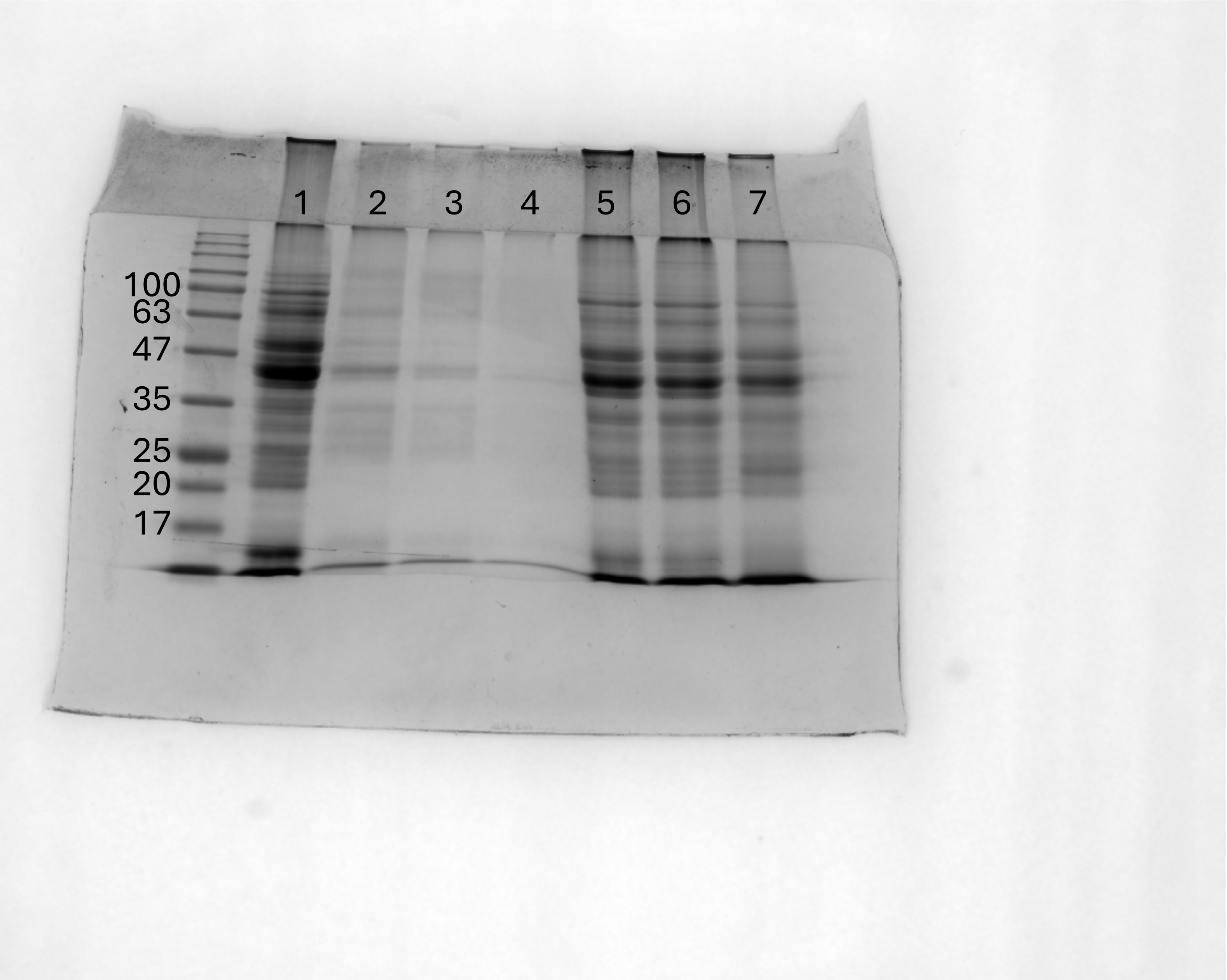

Visualization of soluble fraction by Coomassie brilliant blue

1. Unfixed tissue
2. 1% PFA fixative
3. 2% PFA fixative
4. 3% PFA fixative
5. 1% glyoxal
6. 2% glyoxal
7. 3% glyoxal

Figures 7D

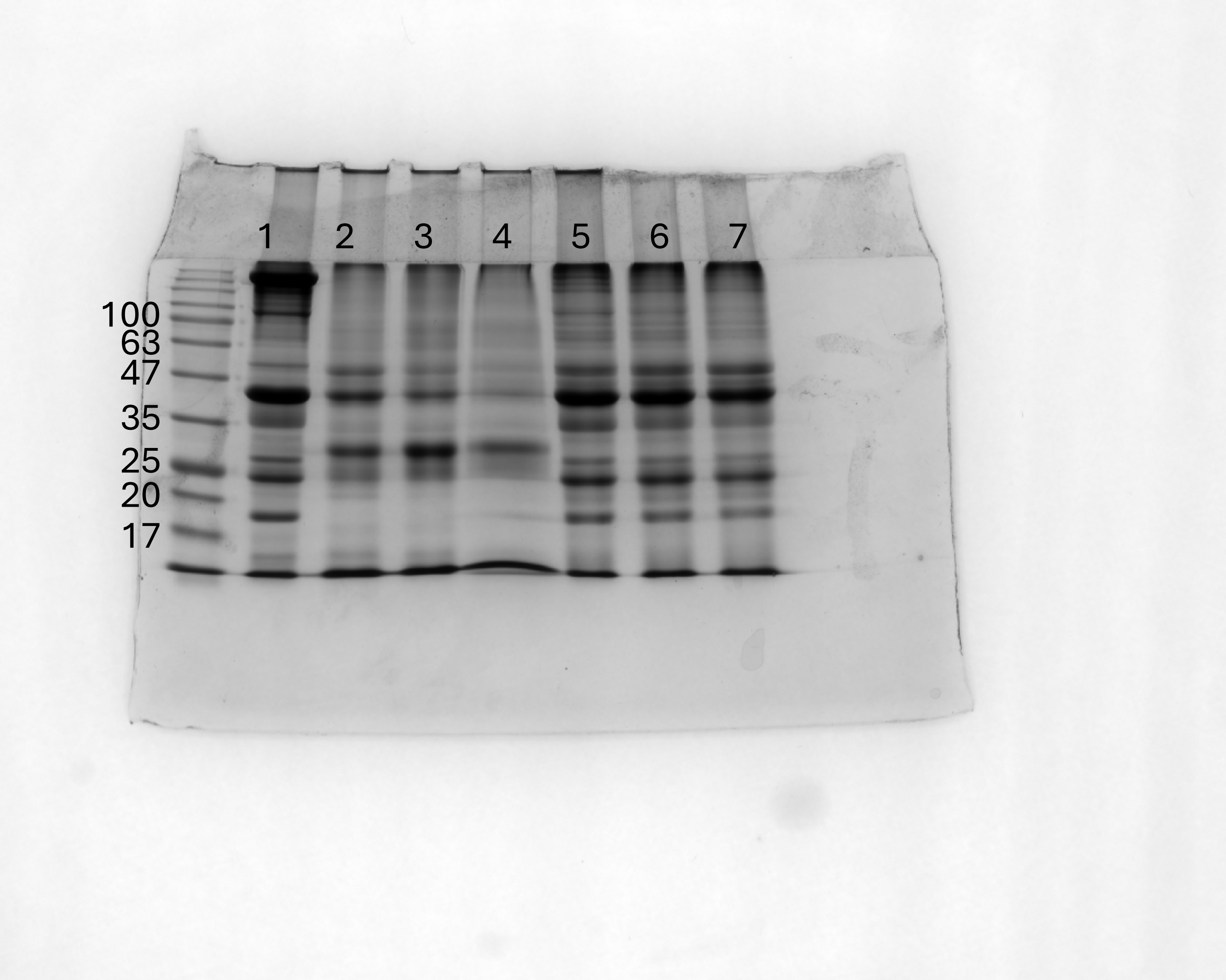

Visualization of insoluble fraction by Coomassie brilliant blue

1. Unfixed tissue
2. 1% PFA fixative
3. 2% PFA fixative
4. 3% PFA fixative
5. 1% glyoxal fixative
6. 2% glyoxal fixative
7. 3% glyoxal fixative

Figure 7F (PLN)

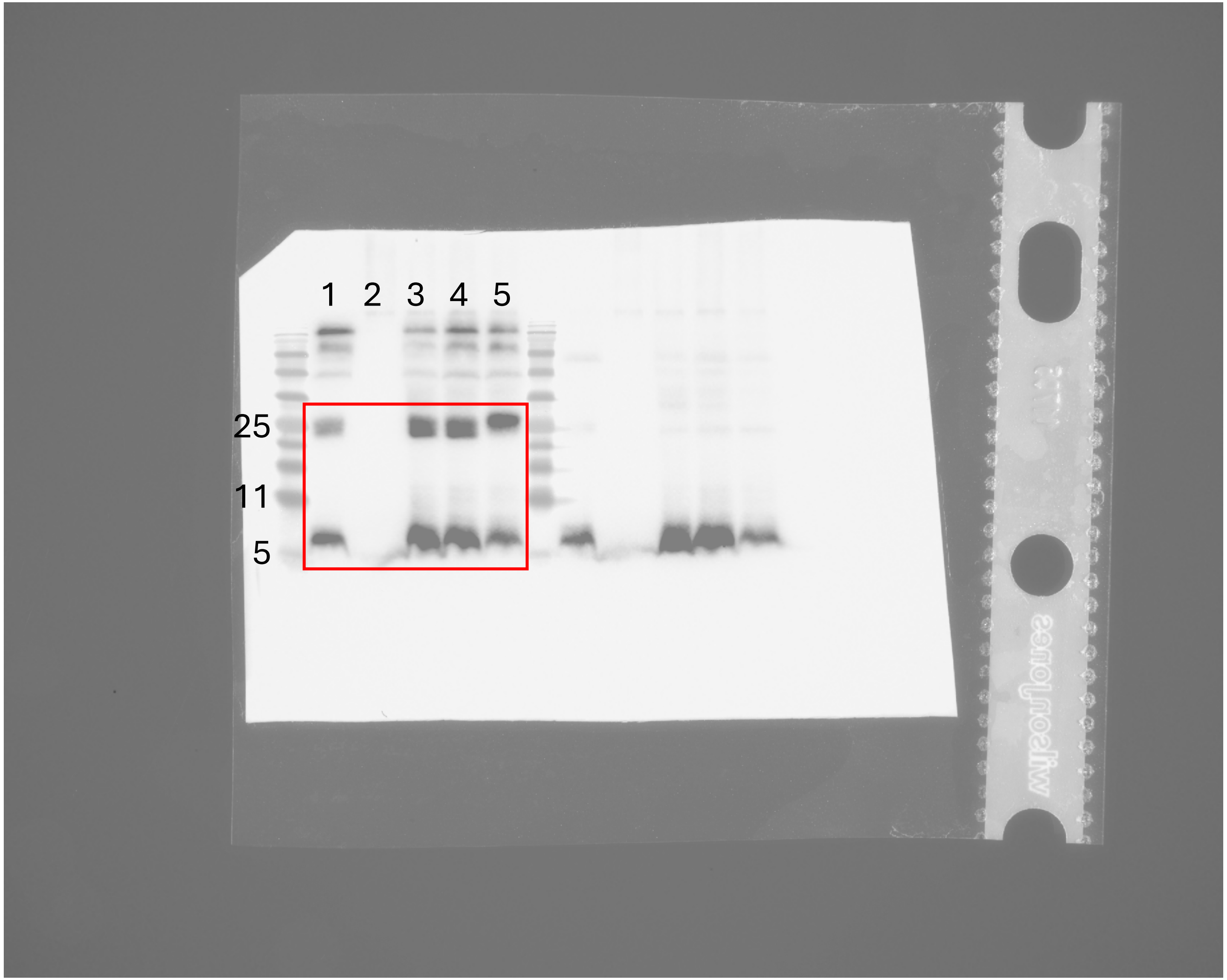

PLN immunoblotting (5 and 25 kDa)

1. Unfixed tissue
2. 4% PFA fixative
3. 1% glyoxal fixative
4. 2% glyoxal fixative
5. 3% glyoxal fixative

Figure 7F (SERCA2)

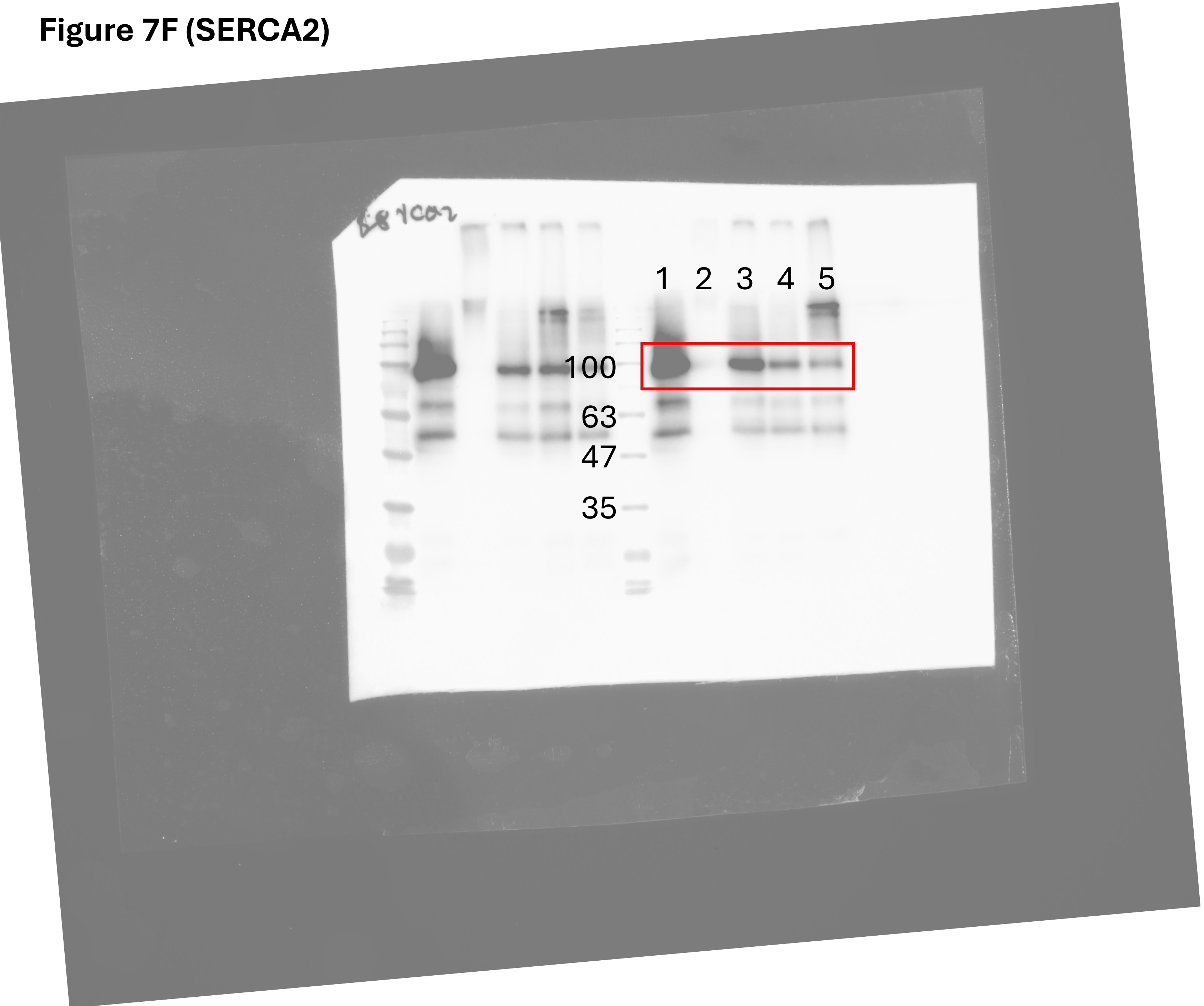

PLN immunoblotting (120 kDa)

1. Unfixed tissue
2. 4% PFA fixative
3. 1% glyoxal fixative
4. 2% glyoxal fixative
5. 3% glyoxal fixative

Figure 7F (alpha-Actin)

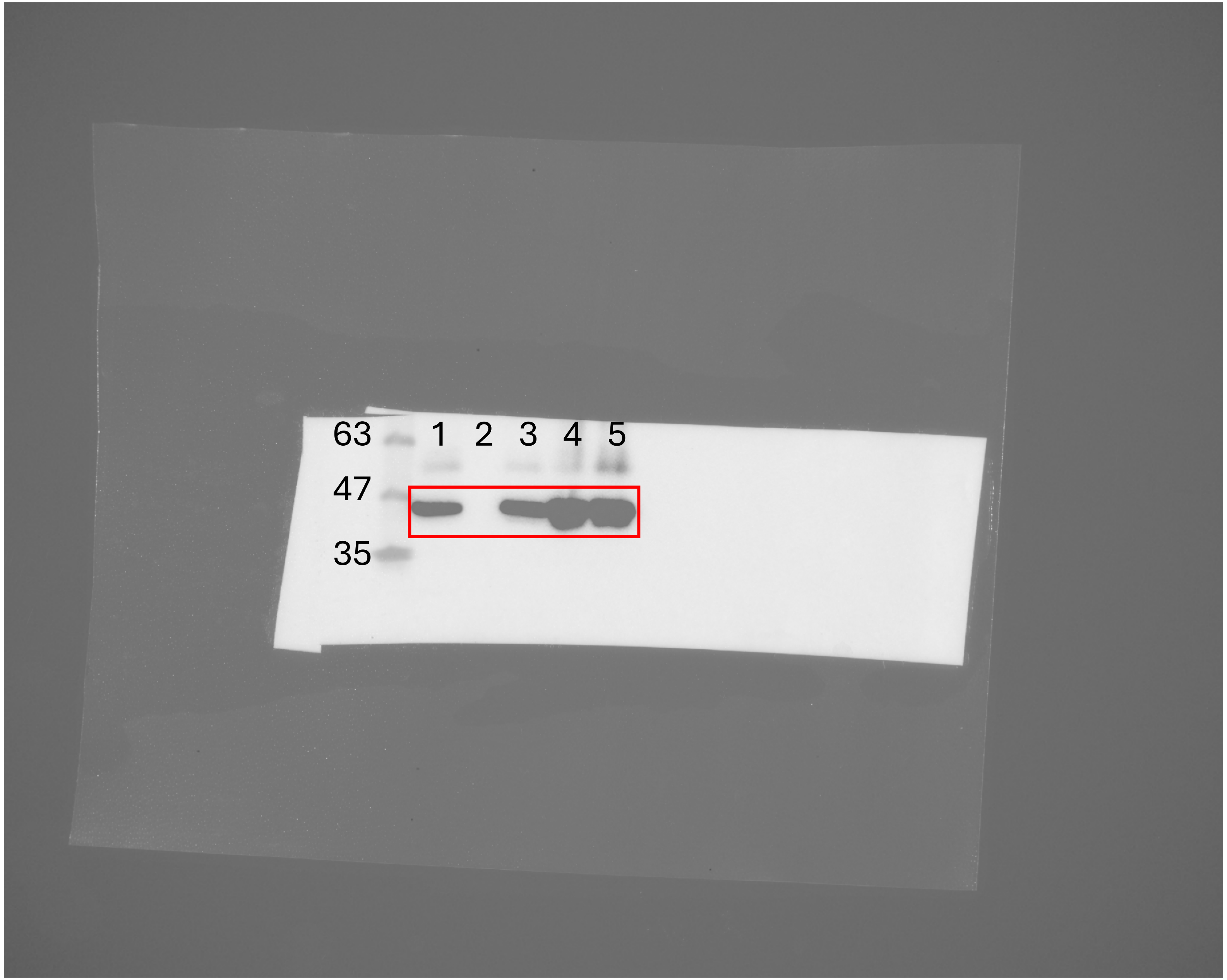

Alpha-actin immunoblotting (37 kDa)

1. Unfixed tissue
2. 4% PFA fixative
3. 1% glyoxal fixative
4. 2% glyoxal fixative
5. 3% glyoxal fixative

Figure 7F (Cx43)

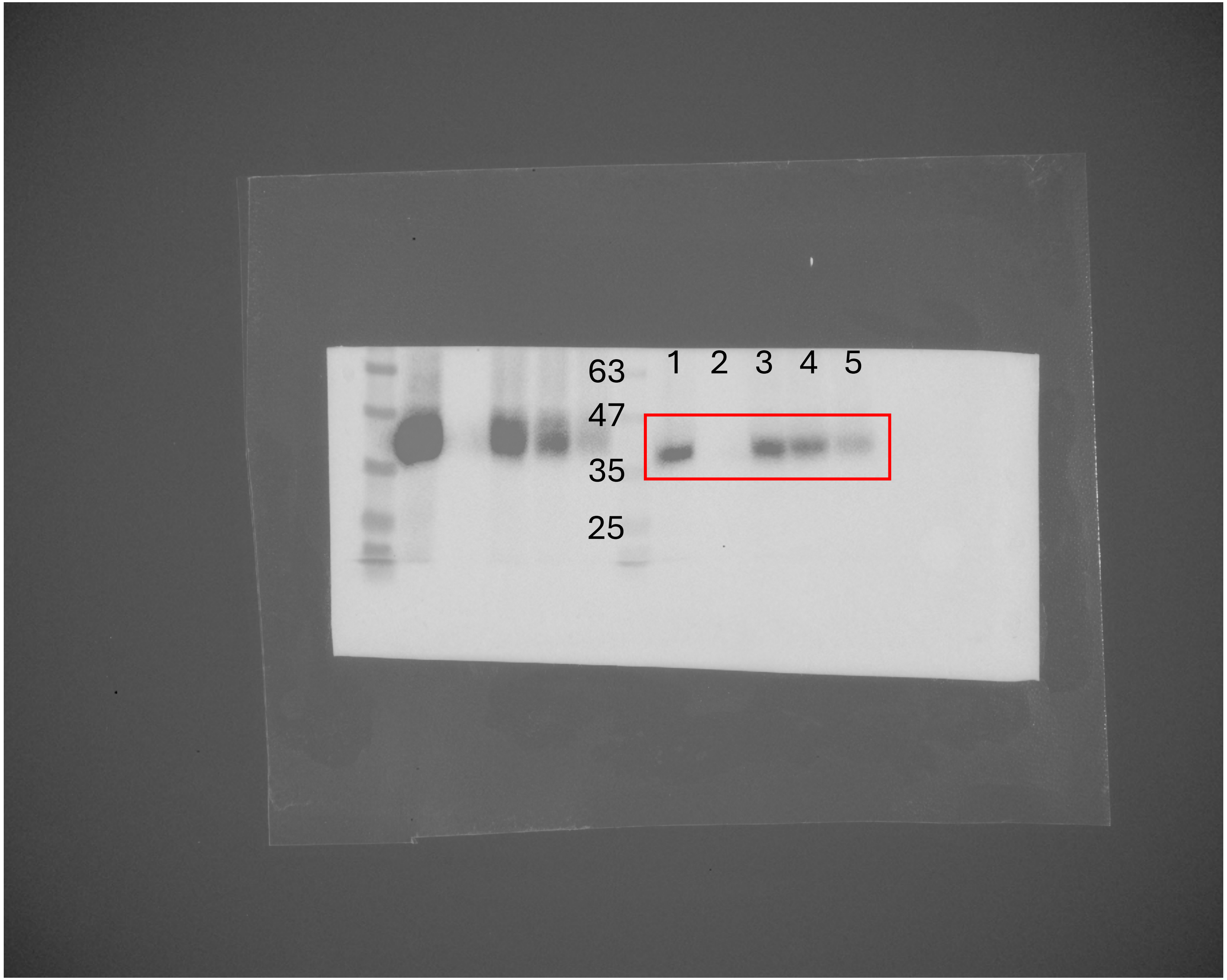

Cx43 immunoblotting (43 kDa)

1. Unfixed tissue
2. 4% PFA fixative
3. 1% glyoxal fixative
4. 2% glyoxal fixative
5. 3% glyoxal fixative

Figure 7F (N-Cad)

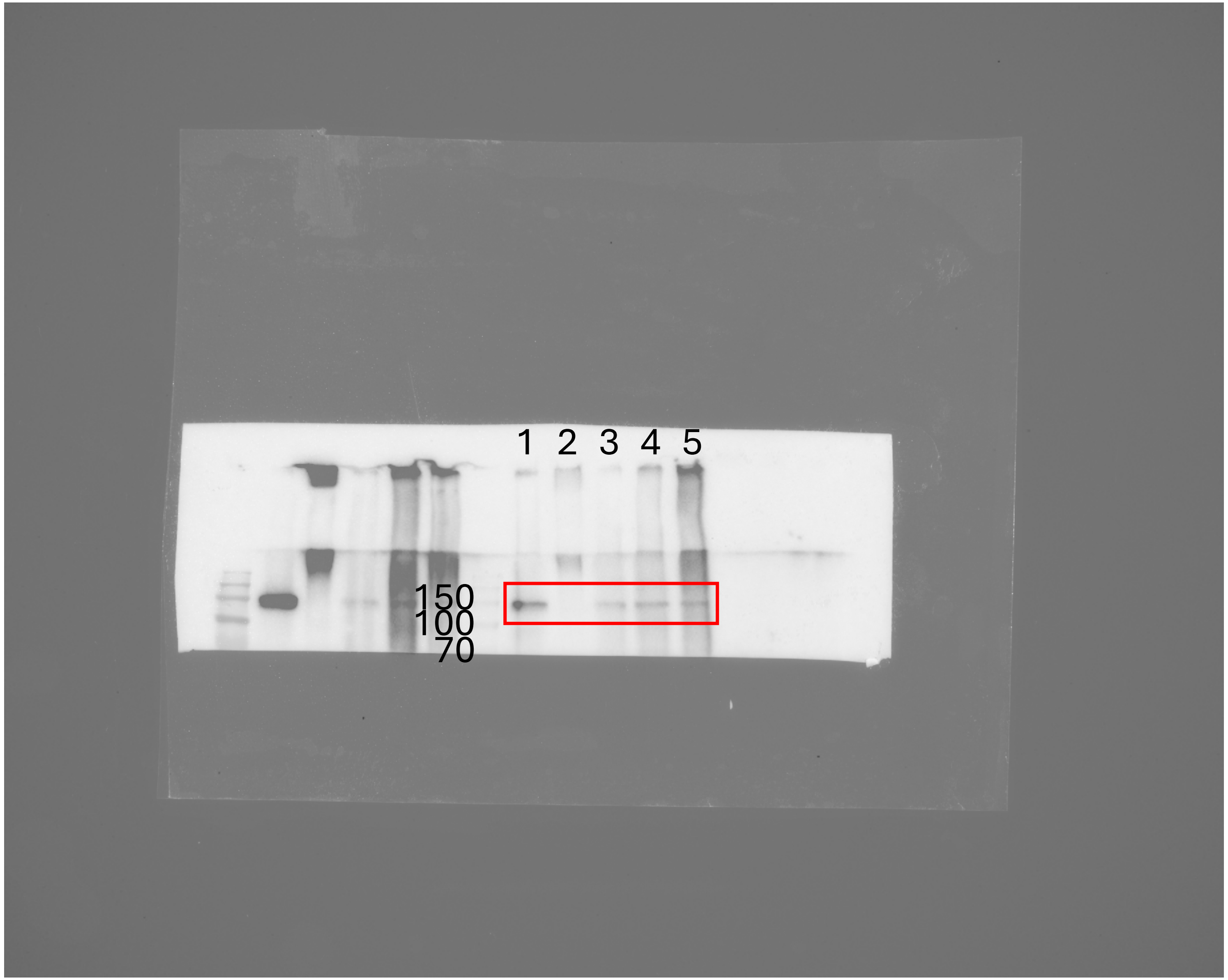

N-Cad immunoblotting (140 kDa)

1. Unfixed tissue
2. 4% PFA fixative
3. 1% glyoxal fixative
4. 2% glyoxal fixative
5. 3% glyoxal fixative

Figure 8A

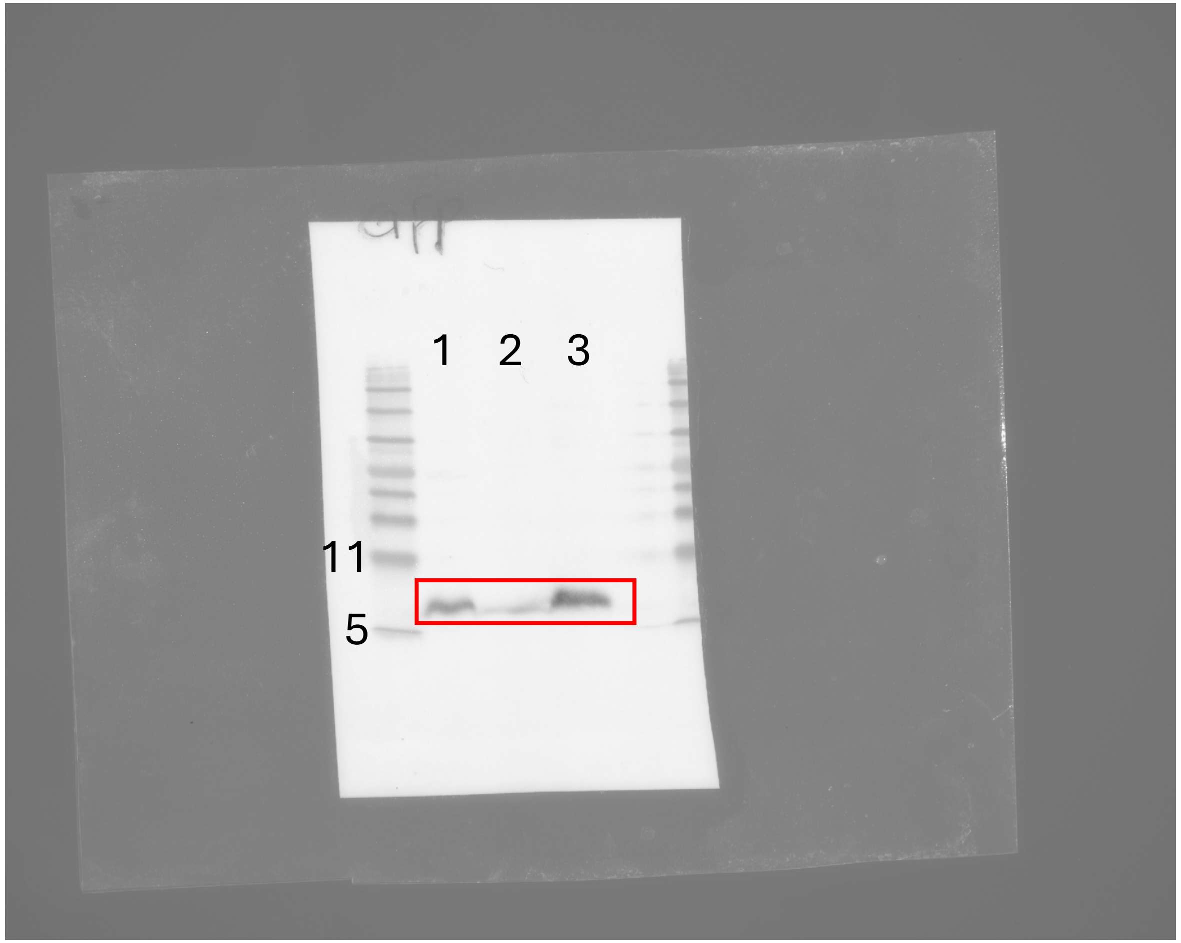

PLN immunoprecipitation from mouse hearts

1. Input (20%)
2. Mock IP (No antibody)
3. IP with PLN antibody

Figure 8E. PLN-ITGB1 co-IP

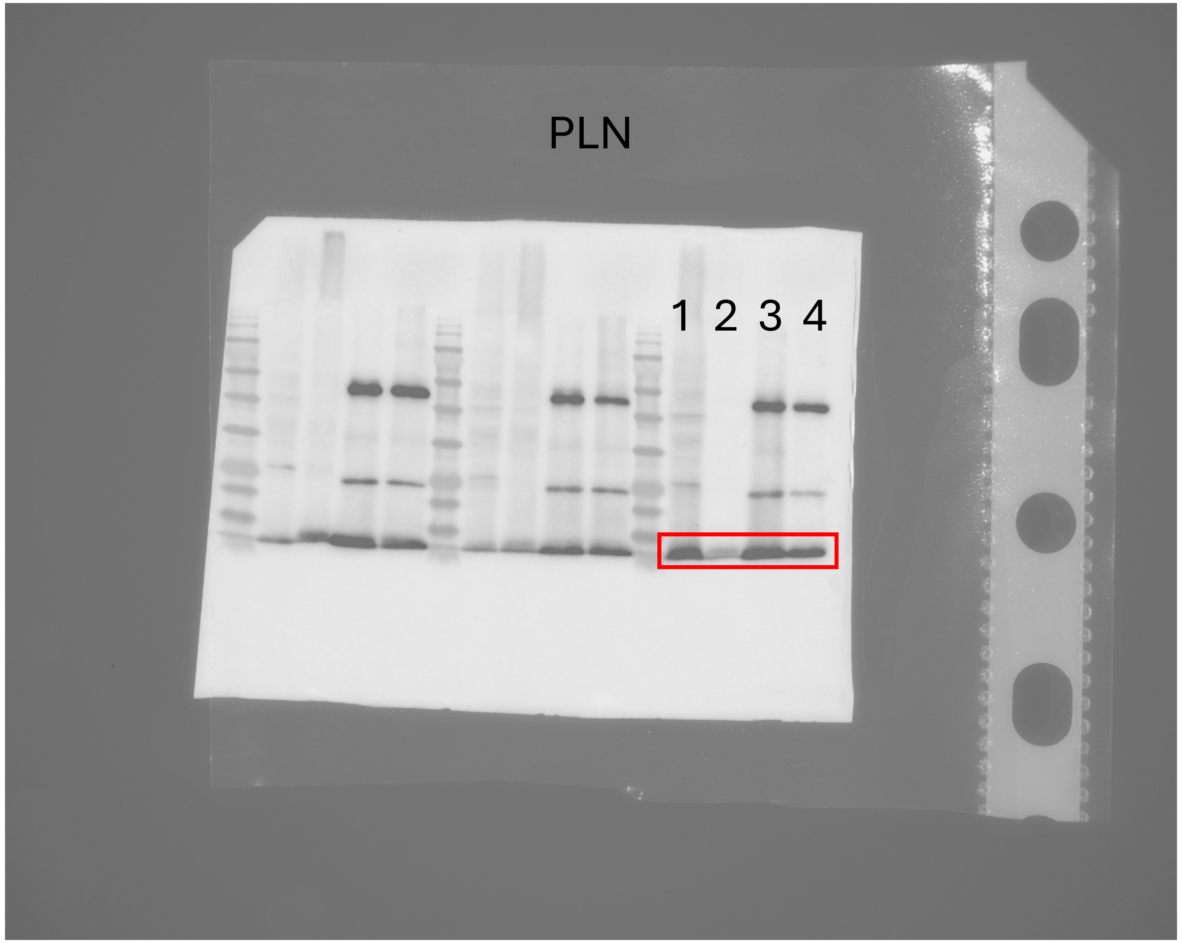

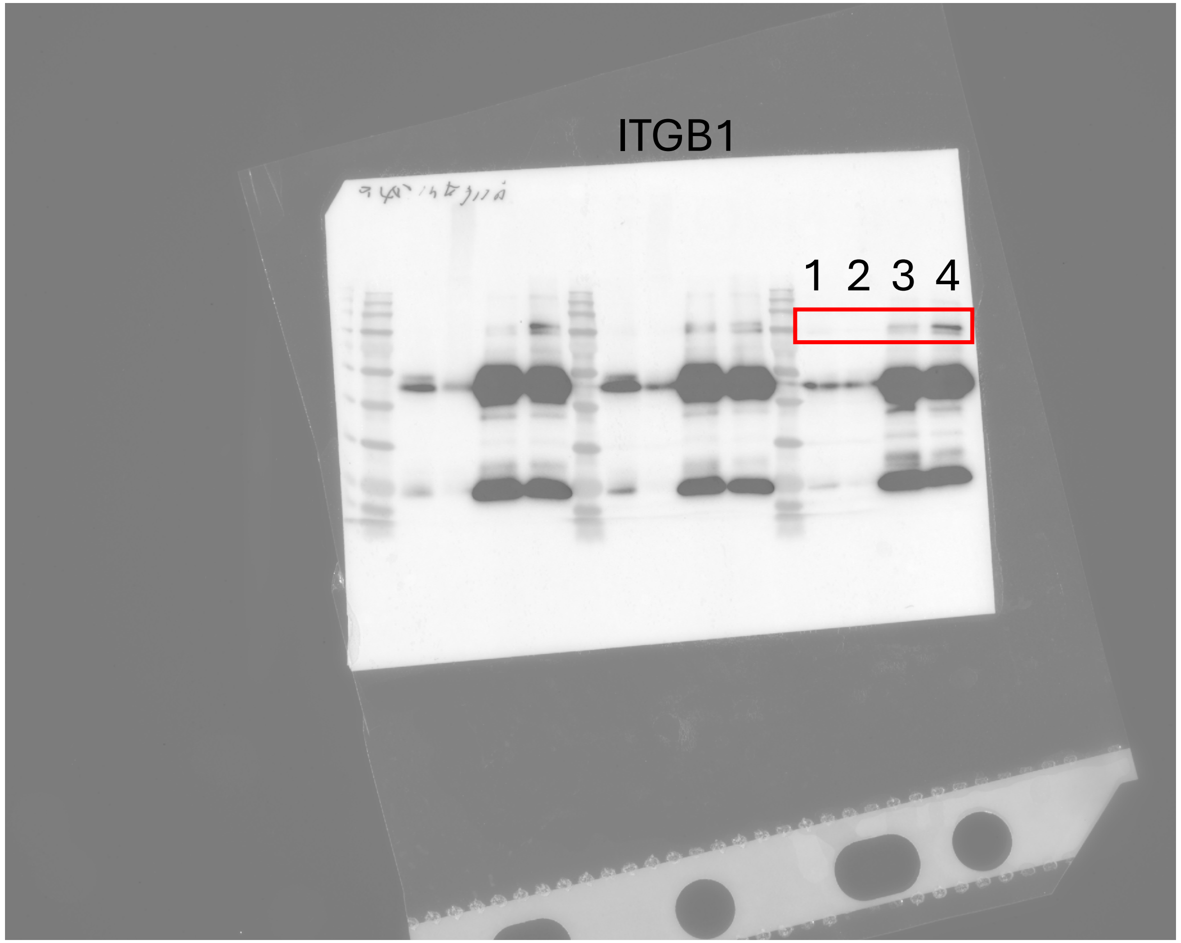

Co-IP from mouse hearts

1. Input, unfixed cardiac lysate (20%)
2. Input, glyoxal fixed cardiac lysate (20%)
3. PLN IP with unfixed cardiac lysate
4. PLN IP with glyoxal-fixed cardiac lysate

Figure 8E. PLN-SERCA2 co-IP

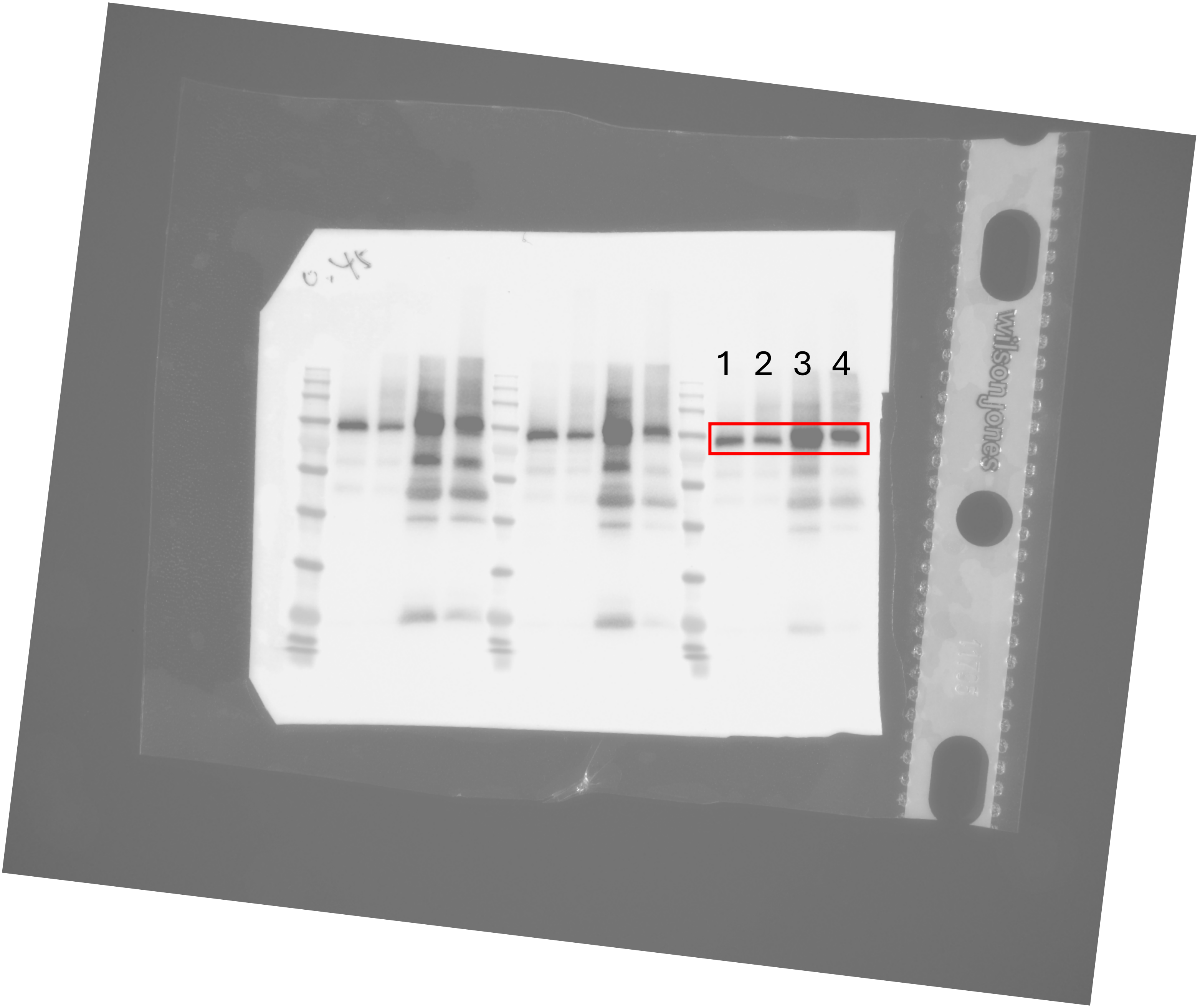

IB SERCA2

1. Input, unfixed cardiac lysate (20%)
2. Input, glyoxal fixed cardiac lysate (20%)
3. PLN IP with unfixed cardiac lysate
4. PLN IP with glyoxal-fixed cardiac lysate

Figure 8E. PLN-Cx43 co-IP

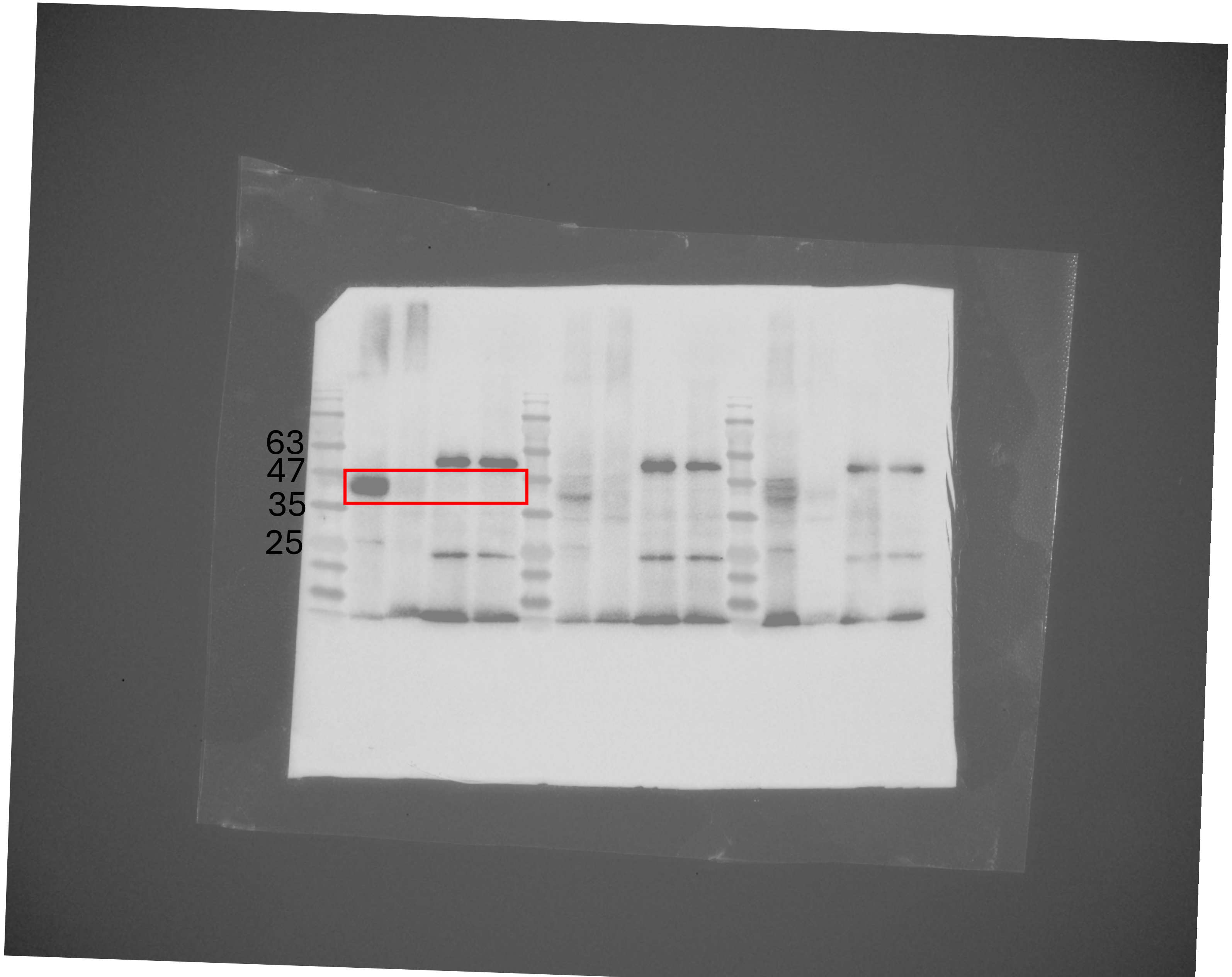

IB SERCA2

1. Input, unfixed cardiac lysate (20%)
2. Input, glyoxal fixed cardiac lysate (20%)
3. PLN IP with unfixed cardiac lysate
4. PLN IP with glyoxal-fixed cardiac lysate

Figure 8E. PLN-alpha ACTIN co-IP

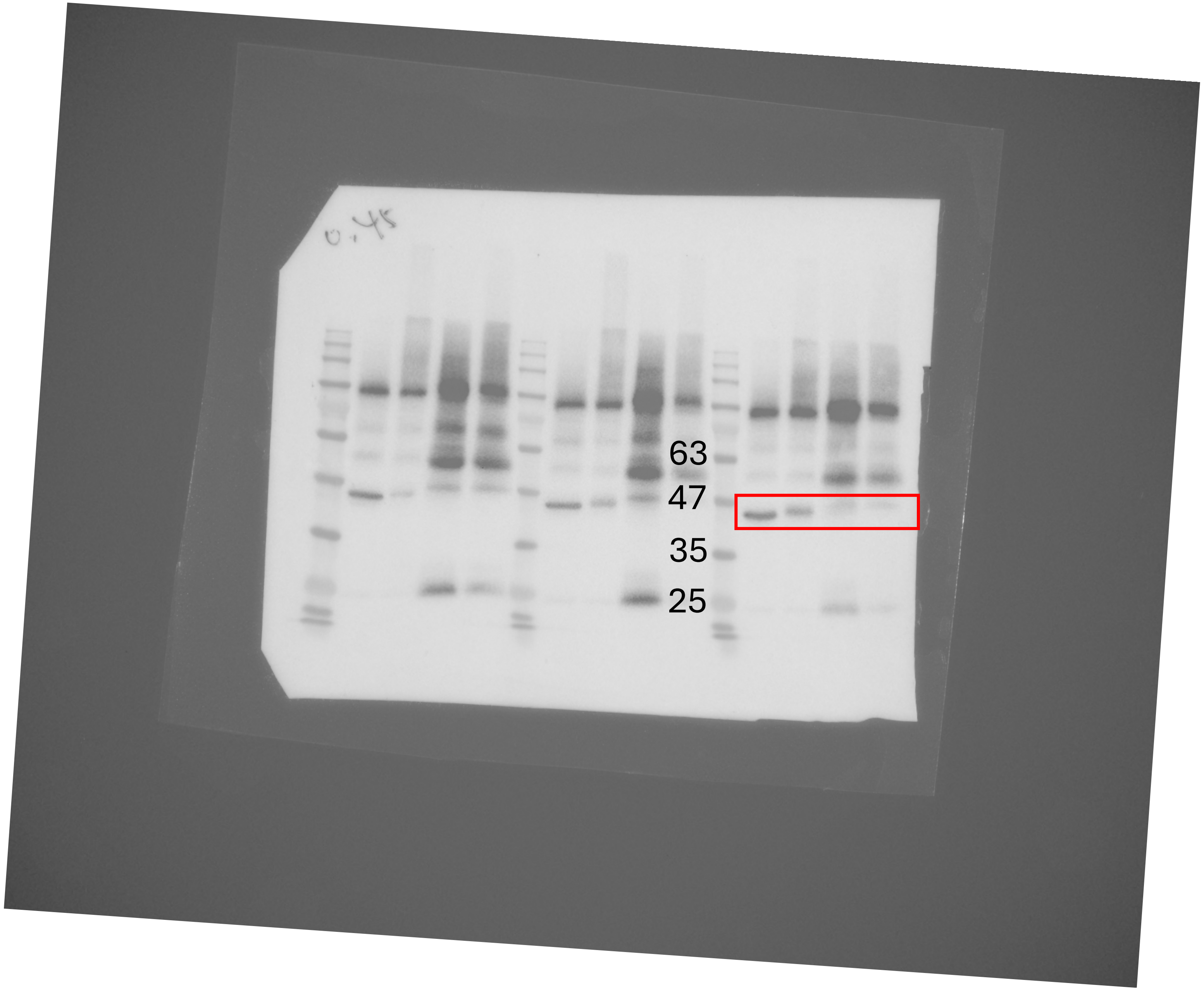

IB SERCA2

1. Input, unfixed cardiac lysate (20%)
2. Input, glyoxal fixed cardiac lysate (20%)
3. PLN IP with unfixed cardiac lysate
4. PLN IP with glyoxal-fixed cardiac lysate
